# Bedaquiline, an FDA-approved drug, inhibits mitochondrial ATP production and metastasis in vivo, by targeting the gamma subunit (ATP5F1C) of the ATP synthase

**DOI:** 10.1038/s41418-021-00788-x

**Published:** 2021-05-13

**Authors:** Marco Fiorillo, Cristian Scatena, Antonio Giuseppe Naccarato, Federica Sotgia, Michael P. Lisanti

**Affiliations:** 1 Translational Medicine, School of Science, Engineering and Environment (SEE), University of Salford, Greater Manchester, UK; 2 The Department of Pharmacy, Health and Nutritional Sciences, The University of Calabria, Cosenza, Italy; 3 Division of Pathology, Department of Translational Research and New Technologies in Medicine and Surgery, University of Pisa, Pisa, Italy; 4 Department of Laboratory Medicine, Pisa University Hospital, Anatomia Patologica 1 Universitaria, Pisa, Italy

**Keywords:** Metastasis, Tumour heterogeneity

## Abstract

Here, we provide evidence that high ATP production by the mitochondrial ATP-synthase is a new therapeutic target for anticancer therapy, especially for preventing tumor progression. More specifically, we isolated a subpopulation of ATP-high cancer cells which are phenotypically aggressive and demonstrate increases in proliferation, stemness, anchorage-independence, cell migration, invasion and multi-drug resistance, as well as high antioxidant capacity. Clinically, these findings have important implications for understanding treatment failure and cancer cell dormancy. Using bioinformatic analysis of patient samples, we defined a mitochondrial-related gene signature for metastasis, which features the gamma-subunit of the mitochondrial ATP-synthase (ATP5F1C). The relationship between ATP5F1C protein expression and metastasis was indeed confirmed by immunohistochemistry. Next, we used MDA-MB-231 cells as a model system to functionally validate these findings. Importantly, ATP-high MDA-MB-231 cells showed a nearly fivefold increase in metastatic capacity in vivo. Consistent with these observations, ATP-high cells overexpressed (i) components of mitochondrial complexes I–V, including ATP5F1C, and (ii) markers associated with circulating tumor cells (CTCs) and metastasis, such as EpCAM and VCAM1. Knockdown of ATP5F1C expression significantly reduced ATP-production, anchorage-independent growth, and cell migration, as predicted. Similarly, therapeutic administration of the FDA-approved drug, Bedaquiline, downregulated ATP5F1C expression in vitro and prevented spontaneous metastasis in vivo. In contrast, Bedaquiline had no effect on the growth of non-tumorigenic mammary epithelial cells (MCF10A) or primary tumors in vivo. Taken together, our results suggest that mitochondrial ATP depletion is a new therapeutic strategy for metastasis prophylaxis, to avoid treatment failure. In summary, we conclude that mitochondrial ATP5F1C is a promising new biomarker and molecular target for future drug development, for the prevention of metastatic disease progression.

## Introduction

ATP is the universal bioenergetic “currency” of all living cells and tissues, including microorganisms, such as prokaryotic bacteria and eukaryotic yeast(s) [1–3]

In eukaryotes, mitochondrial organelles function as the “powerhouse” of the cell [4–7]. Mitochondria generate a vast amount of ATP via the TCA cycle and oxidative phosphorylation (OXPHOS), while glycolysis contributes a minor amount of ATP [8]. Conversely, mitochondrial dysfunction induces ATP depletion, resulting in autophagy, apoptosis (programmed cell death), and/or necrosis [9]. Thus, we have proposed that ATP-depletion therapy may be a viable strategy for targeting and eradicating even the “fittest” cancer cells [10–13].

In MCF7 breast cancer cells, mitochondrial-driven OXPHOS contributes to 80–90% of ATP production, while glycolysis only contributes the remaining 10-20%, under normoxic conditions [14, 15]. Therefore, like normal cells, cancer cells are highly dependent on mitochondrial ATP production [14, 16]. However, it still remains largely unknown if ATP levels in cancer cells contribute to “stemness” and cell cycle progression, as well as their ability to undergo 3D anchorage-independent growth, a characteristic feature of metastatic spread.

Here, we now show that the induction of ATP depletion in MDA-MB-231 breast cancer cells with Bedaquiline, an FDA-approved drug, was indeed sufficient to prevent spontaneous metastasis in a xenograft model, without affecting tumor growth, or inducing significant toxicity. Moreover, we demonstrate that Bedaquiline specifically targets ATP5F1C, the gamma-subunit of the mitochondrial ATP-synthase. These findings are consistent with the idea that ATP is a functional biomarker of metastasis.

## Materials and methods

### Bioinformatic analysis

Unbiased label-free proteomics, comparing 2D-monolayers and 3D-mammospheres, was carried out, as previously described, using MCF7 and T47D breast cancer cell lines [17]. Informatics analysis was also performed using a variety of publicly available of GEO DataSets (GSE36953; GSE2034; GSE59000; and GSE55470), archived in the NCBI database, related to 3D growth, primary tumors, metastasis and circulating tumor cells (CTCs). Gene expression profiling data was extracted from these GEO DataSets. HeatMaps were generated with QIAGEN OmicSoft Suite Software. Volcano plots were produced by examining the annotations present in OncoLand Metastatic Cancer (QIAGEN OmicSoft Suite). In addition, we performed functional “core analyses” using Ingenuity Pathway Analysis Software (IPA; QIAGEN), on annotated genes. Gene co-expression profiles were extracted from The Metastatic Breast Cancer Project Provisional (2020), using cBioPortal (https://www.cbioportal.org/); mRNA expression profiling (RNA Seq V2 RSEM) was carried out via RNA-sequencing of breast cancer tissue samples derived from 146 patients, with metastatic disease. See *Supplemental Information and related Figure Legends* for specific details.

### IHC staining with ATP5F1C antibodies

The study included 12 female patients with a diagnosis of invasive breast carcinoma, treated at the University Hospital of Pisa between 2010 and 2020. Among these, the primary tumor and the corresponding axillary lymph nodal metastasis were retrieved in five patients; distant metastasis alone was retrieved in six patients (two bone metastases, two ovarian metastases, one skin metastasis, one brain metastasis); the primary tumor, the corresponding axillary lymph nodal metastasis together with the skin and the liver metastases were retrieved in 1 patient. All investigations were conducted according to the principles expressed in the Declaration of Helsinki; this study was approved by the local Ethical Committee (Prot. n: 17770. Approval date: 28/10/2020). Written consent was obtained from each participant. All retrieved formalin-fixed, paraffin-embedded samples displayed enough tissue to conduct both analyses and storage. Hematoxylin and eosin (H&E) stained slides were reviewed by one pathologist with expertise in breast cancer (CS) and one representative paraffin block was selected. Immunohistochemistry (IHC) for ATP5F1C was carried out from each paraffin block, applying the specific antibody (clone 2A1AA11, Mouse Monoclonal, Abcam; dilution: 1:100, citrate buffer antigen retrieval: pH 6, 8 min; antibody incubation time: 44 min) in an automated immunostainer (BenchMark Ultra, Ventana Medical Systems). ATP5F1C positivity was expressed as intensity of cytoplasmic staining (faint; weak; moderate; strong). All stained sections were evaluated independently and blindly by two pathologists with expertise in breast cancer (CS and AGN). Discrepancies were resolved via consensus on a double-headed microscope.

### Kaplan–Meier (K–M) analysis

To perform K–M analysis on ATP5F1C, we used an open-access online survival analysis tool to interrogate publicly available microarray data from up to 3951 breast cancer patients [18]. For this purpose, we primarily analyzed data from ER(+) patients. Biased array data were excluded from the analysis. This allowed us to identify ATP5F1C (also known as ATP5C1), as a significant prognostic marker. Hazard-ratios were calculated, at the best auto-selected cut-off, and p-values were calculated using the Log-rank test and plotted in R. K–M curves were generated online using the K–M plotter (as high-resolution TIFF files), using univariate analysis: https://kmplot.com/analysis/index.php?p=service&cancer=breast. This approach allowed us to directly perform in silico validation of ATP5F1C as a marker of tumor recurrence (relapse-free survival) and distant metastasis (distant metastasis-free survival). The latest 2020 version of the database was utilized for all these analyses.

### Reagents and model cell lines

BioTracker ATP-Red 1 (#SCT045) was obtained commercially from Merk-Millipore, Inc. BioTracker green fluorescent probes for cystine uptake (#SCT047), gamma-glutamyl-transferase activity (GGT; #SCT028), pluripotent stem cells (#SCT029), hypoxia (#SCT033) and beta-galactosidase (#SCT025), were all obtained from either Sigma Aldrich or Merk-Millipore, Inc. 4-OH-Tamoxifen, Gemcitabine, DPI (Diphenyleneiodonium chloride), Doxycycline and Palbociclib were all purchased from Sigma-Aldrich, Inc. CSC markers were purchased, as follows: (i) CD44 antibodies (APC Mouse Anti-Human CD44, BD Pharmingen, Inc.) and (ii) AldeRed^™^ reagent dye from ALDH Detection Assay (Merk-Millipore, Inc.) for measuring ALDH activity. The Muse® Autophagy LC3-Antibody Based Kit (Luminex, Corp.) was employed for measuring the Autophagy Induction ratio. Reagents for profiling the intracellular levels of metabolites (ATP, GSH, NADH and NADPH) after cell lysis, were all obtained from Promega, Inc. ER(+) [MCF7 and T47D] and triple-negative [MDA-MB-231 and MDA-MB-468] human breast cancer cell lines were obtained from the American Type Culture Collection (ATCC). MCF-10A cells, a non-tumourigenic human breast epithelial cell line, were also obtained from ATCC. All cell lines used were tested using the MycoFluor™ Mycoplasma Detection Kit (Thermo Scientific, Inc.), confirming that they were mycoplasma-free. MCF7, MDA-MB-231, and MDA-MB-48 cells were cultured in DMEM (High Glucose) supplemented with 10% Fetal Bovine Serum (FBS, Sigma Aldrich), 2 mM Glutamax (Gibco, Life Technologies, Waltham, MA, USA), and 1% penicillin/streptomycin (Gibco, Life Technologies). DMEM/F12 (Sigma Aldrich) supplemented with 10% FBS, 2 mM Glutamax, and 1% penicillin/streptomycin was used to culture T47D cells. Analysis and treatments were performed in the above-mentioned media. The MCF10A cell line was maintained in a mammary epithelial cell growth medium (MEGM; Lonza, Basel, Switzerland) supplemented with 0.4% Bovine pituitary extract, 0.1% insulin, 0.1% hEGF, 0.1% Hydrocortisone, 0.1% GA-1000, and 100 ng/mL of cholera toxin. All cell lines were cultured at 37  °C in 5% CO_2_ in a humidified atmosphere.

### Cell sorting and flow cytometry after vital staining with BioTracker ATP-red 1

Human breast cancer cell lines were first grown either as a 2D monolayer or as 3D-mammospheres. Then, they were stained with 10 µM of BioTracker ATP-Red 1 for at least 30 min. The cells were washed twice with Dulbecco’s phosphate-buffered saline (DPBS; Gibco, Life Technologies), collected and dissociated into a single-cell suspension, using a 25-g needle syringe and a 40 µm cell strainer (Fisher Scientific, Inc.), prior to analysis or sorting by flow-cytometry with the SONY SH800 Cell Sorter [19]. Briefly, ATP-high and ATP-low subpopulations of cells were isolated after vital staining with the probe ATP-Red 1. The ATP-high and ATP-low cell subpopulations were selected by gating, within the ATP-Red 1 signal (Excitation: 510 nm; Emission: 570 nm). Only cells with the least (bottom 5 or 10%) or the most (top 5 or 10%) signal were collected. Also, a population between the bottom and the top populations was sorted (bulk 5%). The cells outside the gates were discarded during sorting, due to the gate settings. However, such settings are required, to ensure high purity during sorting. Data were analyzed with FlowJo 10.1 software.

### Fluorescence imaging

After 24 h post-sorting, all the cell lines seeded in black 96-well plates were stained with 16 µM of Hoescht 33342 (Thermo Fischer, Inc.) and 10 µM of BioTracker ATP-Red 1. Then, the cells were washed twice with DPBS and the images were acquired at 20× magnification, using the EVOS FL 2 automated microscope.

### Flow cytometry after staining with BioTracker fluorescent probes

MCF7 cells first grown as a 2D-monolayer, were stained with BioTracker fluorescent probes for cystine uptake (#SCT047), gamma-glutamyl-transferase activity (GGT; #SCT028), pluripotent stem cells (#SCT029), hypoxia (#SCT033), and beta-galactosidase (#SCT025), respectively, following the concentrations and incubation times, as described by the supplier. Then, the cells were washed twice in PBS, collected and dissociated into a single-cell suspension, using a 40 µm cell strainer. The cells were sorted using the SONY SH800 Cell Sorter. Briefly, probe-high and probe-low subpopulations of cells were isolated. Only cells with the least (bottom 5%) or the most (top 5%) signal were collected. The cells outside the gates were discarded during sorting, due to the gate settings.

### ATP assay using Cell-Titer-Glo

Cell-Titer-Glo (#G7570) was obtained from Promega, Inc., and was used according to the manufacturer’s recommendations, to measure ATP levels in lysed cells. Cell-Titer-Glo is a luciferase-based assay system. Luminescence content was evaluated using the Varioskan^™^ LUX plate reader.

### ATP assay using BioTracker ATP-red 1

Cells were stained with BioTracker ATP-Red 1 after various treatments. After 30 min of incubation, the cells were washed twice with DPBS, collected and dissociated into a single-cell suspension with a 40 µm cell strainer. The cells were analyzed using the Attune NxT Flow Cytometer. Means of the signal (at 570 nm) were compared.

### 3D anchorage-independent growth assay

A single-cell suspension was prepared using enzymatic, and manual disaggregation (25-g needle). Then, cells were plated at a density of 500 cells/cm^2^ in mammosphere medium (DMEM-F12 + 1× B-27 Plus Supplement + 20 ng/ml EGF + Pen/Strep) under non-adherent conditions, in culture dishes precoated with (2-hydroxyethylmethacrylate) (poly-HEMA, Sigma Aldrich Inc.), called “mammosphere plates.” Cells were grown for 5 days and maintained in a humidified incubator at 37 °C. After 5 days of culture, 3D-mammospheres >50 μm were counted using an eyepiece (“graticule”), and the percentage of cells plated which formed spheres was calculated and is referred to as percent mammosphere formation, and was normalized to one (1 = 100% mammosphere formation efficiency (MFE)). 3D MFE was analyzed in both the ATP-low and ATP-high subpopulations of cells. All 3D mammosphere experiments were performed in triplicate, at least three times independently.

### 3D spheroid formation assay

3D Spheroids were generated following the application note by Thermo Fisher (https://assets.thermofisher.com/TFS-Assets/BID/Application-Notes/mcf7-spheroids-serum-free-application-note). Cells were seeded in DMEM/F-12, HEPES, no phenol red, and 1× B-27 Plus Supplement, 10 ng/mL of HS bFGF (Gibco Life Technologies) for a total volume of 0.2 mL/well and a total of 500 cells/well. The plate was then centrifuged at 120 × *g* for 5 min before being placed in a 37 °C, 5% CO_2_ incubator. The spheroids were incubated for 10 days without changing the media before analysis, but the spheroids were observed to form overnight after seeding.

### MCF7 cells harboring an ATP-binding protein biosensor

Tempo-ATP-MCF7 cells, recombinantly over-expressing a cytosolic fluorescent protein ATP-biosensor, were custom-generated by Tempo-Bioscience, Inc. (San Francisco, CA, USA), using a puromycin-resistance marker for cell selection. This protein-based fluorescent ATP-biosensor has an excitation wavelength of 517–519 nm and an emission of 535 nm. It consists of an ATP-binding peptide (MDYKDDDDKKTNWQKRIYRVKPCVICKVAPRDWWVENRHLRIYTMCKTCFSNCINYGDDTYYGHDDWLMYTDCKEFSNTYHNLGRLPDEDRHWSASCHHHHHHMGMSGS) [20] fused in-frame with a GFP-like fluorescent reporter protein.

### shRNA lentiviral transduction

Lentiviral plasmids, packaging cells, and reagents were purchased from Genecopoeia. Forty-eight hours after seeding, 293Ta packaging cells were transfected with lentiviral vectors encoding an shRNA clone set of three constructs against all three variants for human ATP5F1C, in a lentiviral psi-LVRInU6TGP vector, with an inducible U6 promoter, CMV promoter-TetR-SV40 promoter-eGFP-IRES-puromycin. A scrambled control psi-LVRInU6TGP vector (sh-Control) was transfected in parallel. Two days post-transfection, the lentivirus-containing culture medium was passed through a 0.45 µm filter and added to the target cells (MDA-MB-231), in the presence of 5 µg/ml Polybrene. Infected cells were selected, with a concentration of 1.5 µg/ml of puromycin.

### Western blotting

Cells were lysed in RIPA buffer (Sigma Aldrich, Inc.) containing one tablet of Complete^TM^ inhibitor mix (Roche Applied Science, Indianapolis) and one tablet of PhosSTOP^™^ phosphate inhibitors per 10 mL of buffer and loaded onto SDS-polyacrylamide gels. The gels were transferred to 0.2 µm nitrocellulose membranes, using the Trans-Blot Turbo Transfer System (Bio-Rad, Inc.) Membranes were incubated with the respective primary antibodies diluted in Tris-buffered saline, 0.1% Tween 20 (Sigma Aldrich, Inc.) and 5% bovine serum albumin (BSA; Sigma Aldrich Inc.) (TBST), and incubated overnight at 4 °C. Then, the blots were washed and incubated with appropriate secondary antibodies and detected using Super Signal West Pico Chemiluminescent Substrate (Thermo Scientific, Inc.), using the G-Box (Syngene, Inc). Antibodies and their dilutions used for Western blot analysis were as follows: mouse anti-ATP5F1C 1:500, mouse anti-HSP60 1:1000, mouse anti-HIF-1α 1:500, mouse anti-human total OXPHOS cocktail 1:1000, mouse anti-Ep-CAM 1:500, mouse anti-VCAM-1 1:500, mouse anti-β-tubulin 1:2000, mouse anti-β-actin 1:10,000, rabbit anti-PARP 1:1000, rabbit anti-p21 1:1000, rabbit anti-SQSTM1/p62 1:1000, and mouse anti-Vinculin 1:1000. The resulting immunoblot images were acquired using GeneSys Software (Syngene, Inc.)

### Metabolic flux analysis with the Seahorse XFe96

Real-time oxygen consumption rates (OCRs) and extracellular acidification rates (ECARs) rates were determined using the Seahorse Extracellular Flux (XFe96) analyzer (Seahorse Bioscience, USA) [21, 22]. Briefly, 3 × 10^4^ cells per well were seeded into XFe96 well cell culture plates after sorting and incubated for 12 h to allow cell attachment. After 24 h of incubation, cells were washed in pre-warmed XF assay media (or for OCR measurement, XF assay media supplemented with 10 mM glucose, 1 mM Pyruvate, 2 mM l-glutamine, and adjusted at 7.4 pH). Cells were then maintained in 175 μL/well of XF assay media at 37 °C, in a non-CO_2_ incubator for 1 h. During the incubation time, we loaded 25 μL of 80 mM glucose, 9 μM oligomycin, and 1 M 2-deoxyglucose (for ECAR measurement) or 10 μM oligomycin, 9 μM FCCP, 10 μM rotenone, 10 μM antimycin A (for OCR measurement), in XF assay media into the injection ports in the XFe96 sensor cartridge. Measurements were normalized by Hoechst 33342 content and/or protein content (SRB assay). Data sets were analyzed using XFe96 software and GraphPad Prism software, using one-way ANOVA and Student’s *t* test calculations. All experiments were performed in quintuplicate, three times independently.

### Cell cycle analysis by fluorescence-activated cell sorting (FACS)

We performed cell-cycle analysis on the ATP-high and ATP-low cell subpopulations, by FACS analysis using the Attune NxT Flow Cytometer. Briefly, after trypsinization, the resuspended cells were incubated with propidium iodide, as per the manufacturer’s recommendations (Cell Cycle Kit; Merck Millipore, Inc.). We analyzed 25,000 events per condition. Gated cells were categorized into cell-cycle stages.

### Cell migration and invasion assays

Briefly, 3.5 × 10^4^ cells in 0.5 ml of serum-free DMEM with 0.1% BSA were added to the wells of 8-μm pore, non-coated membrane modified Boyden chambers (Transwells). The lower chambers contained 10% FBS in DMEM to serve as a chemo-attractant. Cells were incubated at 37 °C and allowed to migrate throughout the course of 16 h. Noninvasive cells were removed from the upper surface of the membrane by scrubbing with cotton swabs. Chambers were stained in 0.5% crystal violet diluted in 100% methanol for 30–60 min, rinsed in water and examined under a bright-field microscope. Values for invasion and migration were obtained by counting five fields per membrane (20× objective) and represent the average of three independent experiments. Note that Transwells, precoated with extracellular matrix (namely Matrigel), were used to measure aggressive cell invasion and prevent simple cell migration.

### Flow cytometry analysis with LC3 antibodies

The Autophagy Induction ratio was calculated using an autophagy LC3-antibody-based Kit for the Guava® Muse® Cell Analyzer by Luminex. Totally, 5 × 10^4^ MDA-MB-231 cells were treated with Bedaquiline (10 µM) or vehicle alone, for 48, 72, and 120 h, and then subjected to analysis by flow cytometry, following the procedure as described in the kit protocol.

### Metastasis assays

The chick embryo metastasis assay was performed by INOVOTION (Société: 811310127), La Tronche-France. According to the French legislation, no ethical approval is needed for scientific experimentations using oviparous embryos (decree n° 2013-118, February 1, 2013; art. R-214-88). Animal studies were performed under animal experimentation permits N° 381029 and B3851610001 to Jean Viallet (INOVOTION). Fertilized White Leghorn eggs were incubated at 37.5 °C with 50% relative humidity for 9 days. Greater than 20 eggs were processed for each experimental condition. At that moment (E9), the chorioallantoic membrane (CAM) was dropped down by drilling a small hole through the eggshell into the air sac, and a 1 cm^2^ window was cut in the eggshell above the CAM. The MDA-MB-231 tumor cell line was cultivated in DMEM medium supplemented with 10% FBS and 1% penicillin/streptomycin. On day E9, cells were detached with trypsin, washed with complete medium, and suspended in graft medium. After ATP-based cell sorting by flow-cytometry with BioTracker ATP-Red 1, an inoculum of 30,000 cells was added onto the CAM of each egg (E9) and then eggs were randomized into groups. On day E18, a 1 cm^2^ portion of the lower CAM was collected to evaluate the number of metastatic cells in 10 samples per group (*N* = 10). Genomic DNA was extracted from the CAM (commercial kit) and analyzed by qPCR with specific primers for Human Alu sequences. Calculation of Cq for each sample, mean Cq and relative amounts of metastases for each group are directly managed by the Bio-Rad® CFX Maestro software. Non-injected eggs were also evaluated in parallel, as a negative control for specificity. A one-way ANOVA analysis with post-tests was performed on all the data.

### Tumor growth, metastasis, and embryo toxicity assays

Xenograft assays were carried out by INOVOTION (Société: 811310127), La Tronche-France, essentially as we previously described, without any major modifications [23, 24]. Fertilized White Leghorn eggs were incubated at 37.5 °C with 50% relative humidity for 9 days. At that moment (E9), the CAM was dropped down by drilling a small hole through the eggshell into the air sac, and a 1 cm^2^ window was cut in the eggshell above the CAM. The MDA-MB-231 tumor cell line was cultivated in DMEM medium supplemented with 10% FBS and 1% penicillin/streptomycin. On day E9, cells were detached with trypsin, washed with complete medium, and suspended in graft medium. An inoculum of 1 × 10^6^ MDA-MB-231 cells was added onto the CAM of each egg (E9) and then eggs were randomized into groups. On day E10, tumors were detectable, and they were then treated daily for 8 days with vehicle alone (1% DMSO in PBS) or with three different dosages of Bedaquiline. At day 18 (E18), the upper portion of the CAM was removed from each egg, washed in PBS and then directly transferred to paraformaldehyde (fixation for 48 h) and weighed. For tumor growth assays, at least 14 tumor samples were collected and analyzed per group (*N* ≥ 14). On day E18, a 1 cm^2^ portion of the lower CAM was collected to evaluate the number of metastatic cells in at least seven samples per group (*N* ≥ 7). Genomic DNA was extracted from the CAM (commercial kit) and analyzed by qPCR with specific primers for Human Alu sequences. Calculation of Cq for each sample, mean Cq and relative amounts of metastases for each group are directly managed by the Bio-Rad® CFX Maestro software. A one-way ANOVA analysis with post-tests was performed on all the data. Before each administration, the treatment tolerability was evaluated by scoring the number of live and dead embryos.

### Statistical analysis

All analyses were performed with GraphPad Prism 7. Data were represented as mean ± SD (or ±SEM where indicated). All experiments were conducted at least three times independently, with 3 or more technical replicates for each experimental condition tested (unless stated otherwise, e.g., when representative data is shown). Statistically significant differences were determined using the Student’s *t* test or the analysis of variance (ANOVA) test. For the comparison among multiple groups, one-way ANOVA was used to determine statistical significance. *p* < 0.05 was considered significant.

## Results

### Bioinformatic analysis of ATP synthesis and cancer metastasis: Importance of ATP5F1C, the gamma-subunit of the mitochondrial ATP-synthase

Here, we have begun to test the hypothesis that mitochondrial ATP synthesis is a key determinant of 3D anchorage-independent growth and metastasis, using a bioinformatics approach. More specifically, we defined a mitochondrial-related gene signature for metastasis, which features the gamma-subunit of the mitochondrial ATP-synthase (ATP5F1C). Taken together, this analysis is consistent with the hypothesis that increased mitochondrial ATP synthesis could be a key driver of 3D anchorage-independent growth and metastasis. See Supplemental Figs. S1–S6.

**Fig. 1:**
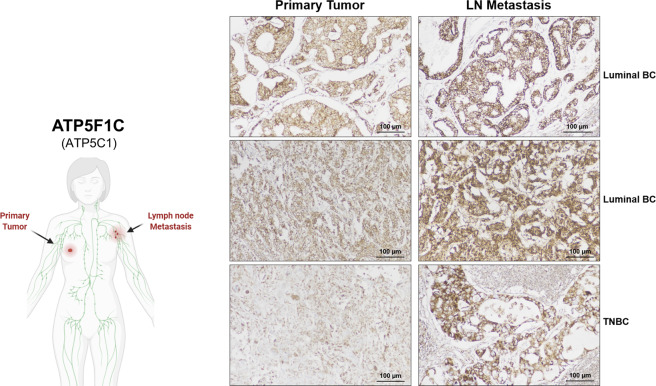
ATP5F1C protein expression is increased in lymph nodal (LN) metastases: immunostaining with monoclonal antibodies directed against ATP5F1C. Matched formalin-fixed paraffin-embedded (FFPE) tissue sections of the primary tumor (Left) and lymph nodal metastases (LN; Right), from the same patients, were subjected to immunohistochemical staining with mono-specific antibodies that recognize ATP5F1C. Note that ATP5F1C expression is increased in lymph node metastases, as compared to the matched primary tumor samples. Three representative examples are shown, from two luminal ER(+) breast cancer (BC) patients and one triple-negative breast cancer (TNBC) patient. Images were acquired with a 20× objective. Scale bar = 100 µm. Please also see Supplemental Fig. S6.

In addition, we validated the protein expression of ATP5F1C in female patients with a diagnosis of invasive breast carcinoma (Luminal ER(+) BC, TNBC, HER2(+) BC). Most notably, ATP5F1C (also known as ATP5C1) expression is increased in lymph-node metastases, as compared to the matched primary tumor samples (Fig. 1). Moreover, ATP5F1C is highly over-expressed in distant metastatic lesions (Fig. S6).

These findings also suggest the attractive idea that mitochondrial ATP levels could confer a proliferative advantage and stem-like traits in cancer cells.

### Bioenergetic fractionation by flow cytometry with BioTracker ATP-Red 1 allows the isolation of cell sub-populations, with differing rates of proliferation

**Fig. 2:**
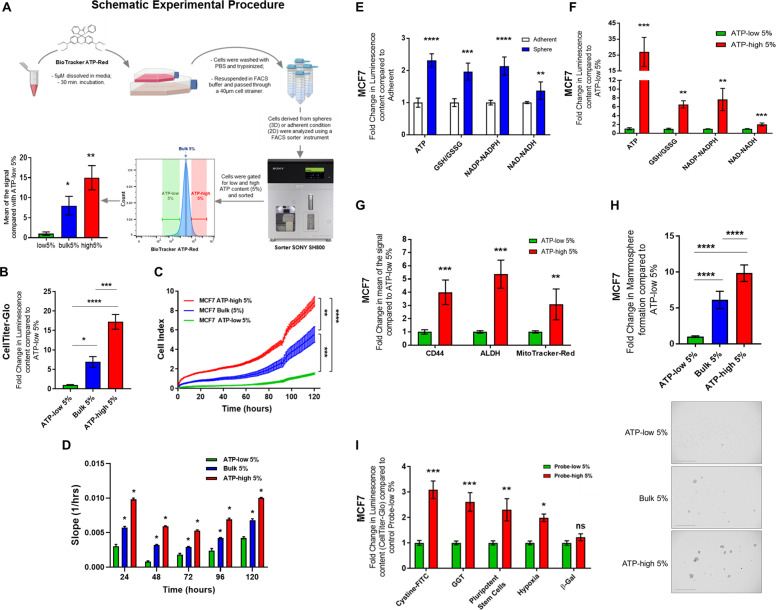
Metabolic cell fractionation with an ATP-binding fluorescent dye reveals that high mitochondrial ATP levels are a key determinant of MCF7 cell proliferation, stemness, 3D anchorage-independent growth and anti-oxidant capacity. Here, we used staining with a vital dye, namely BioTracker ATP-Red 1, to detect ATP levels in living cancer cells and then subjected them to flow cytometry, to isolate the most energetic and the least energetic cancer cells, to compare their phenotypic behavior. Please also see Supplemental Fig. S7. **A** Metabolic fractionation with Biotracker ATP-Red 1. Experimental Procedure summarizing the key steps from live-cell staining to the isolation of the ATP-enriched cell population, using flow cytometry. The Biotracker ATP-Red 1 probe recognizes ATP at the molecular level and becomes fluorescently activated, allowing the detection of mitochondrial ATP levels. Based on mean signal intensity, we estimate that ATP-high MCF7 cells have approximately 15-fold higher levels of ATP, as compared with the ATP-low population; and 2-fold higher levels of ATP, as compared with bulk cell population. Data represent the mean fold increase over ATP-low 5% cells ± SD, *n* = 9. One-way ANOVA, Dunnett’s multiple comparisons test, *p < 0.01, **p < 0.001. **B** Validation of ATP levels after metabolic fractionation. ATP levels in MCF7 cells were determined after flow cytometry, using Cell-Titer-Glo. After cell counting, equal numbers of single cells were then used to evaluate their relative ATP content by luminescence, using the Varioskan^™^ LUX plate reader. Note that ATP-high MCF7 cells showed a >15-fold increase in ATP levels, while bulk cells showed a ~7-fold increase ATP, all relative to ATP-low cell population. ATP-high 5% (*n* = 6), Bulk 5% (*n* = 3), ATP-low 5% (*n* = 6). Data represent the mean fold increase ±SD over ATP-low 5% cells. One-way ANOVA, Dunnett’s multiple comparisons test, *p < 0.05, ***p < 0.0005, ****p < 0.0001. **C** Cell proliferation. Proliferation was assessed using the xCELLigence® RTCA DP instrument. Cells were first sorted for ATP content, counted and seeded (1 × 10^4^ in common media) in RTCA DP E-Plates for real-time growth analysis. Graphically, the three subpopulations (ATP-low 5%, Bulk 5%, ATP-high 5%) are all represented. The results indicate that the ATP-high population is approximately twofold more proliferative, relative to the bulk cell population and approximately 5-fold more proliferative, relative to the ATP-low population, after 120 h. Data represent the mean ± SD, *n* = 3. One-way ANOVA, Dunnett’s multiple comparisons test, **p < 0.001, ***p < 0.001, ****p < 0.0001. **D** Slope analysis of proliferation. Comparative slope analysis of the three cell subpopulations. At each time point (24, 48, 72, 96, and 120 h) the slope of the ATP-high cell population, was significantly higher compared to the other two subpopulations. Data represent mean ± SD, *n* = 3. Two-way ANOVA, Tukey corrected, *p < 0.01. **E** Metabolic analysis of 2D vs. 3D growth. Metabolic analysis of MCF7 2D monolayers and 3D mammospheres. Key metabolite levels in MCF7 cells grown as 2D adherent monolayers or 3D mammospheres were compared, using Promega kits (Cell-Titer-Glo, GSH/GSSG-Glo, NADP-NADPH-Glo, NAD-NADH-Glo). 2D monolayers and 3D mammospheres were first dissociated into single cells with trypsin, syringed with a 25-gauge needle and passed through a 40-µm cell strainer. After cell counting, equal numbers of single cells were then used to evaluate their relative luminescence content. Note that cells derived from 3D mammospheres showed a >2-fold increase in ATP levels; a near 2-fold increase in reduced glutathione levels; a >2-fold increase in NADP-NADPH levels and a near 1.5-fold increase in NAD-NADH levels, all relative to 2D monolayers. Data represent the mean fold increase over adherent cells ±SD, *n* = 4. Unpaired *t* test, **p < 0.005, ***p < 0.0005, ****p < 0.0001. **F** Metabolic analysis of ATP-high vs. ATP low cells. Measurement of ATP, glutathione, NADH, and NADPH were carried out in ATP-high MCF7 cells, as compared with ATP-low MCF7 cells. Key metabolite levels were measured using Promega kits (Cell-Titer-Glo, GSH/GSSG-Glo, NADP-NADPH-Glo, NAD-NADH-Glo). Cells in 2D monolayers were first stained with BioTracker ATP-Red 1 and sorted by ATP content by flow cytometry. After cell counting, equal numbers of single cells were then used to evaluate their relative luminescence content. Note that ATP-high cells showed a near 25-fold increase in ATP levels; a 6-fold increase in reduced glutathione levels; a nearly 8-fold increase in NADP-NADPH levels and >2-fold increase in NAD-NADH levels, all relative to ATP-low MCF7 cells. Data represent the mean fold increase over ATP-low 5% cells ±SD, *n* = 4. Unpaired *t* test, **p < 0.005, ***p < 0.0005. **G** ATP-high cells are enriched in well-established CSC markers, and show elevated mitochondrial mass. Stemness was first examined using two well-established CSC biomarkers, namely CD44 expression and ALDH activity. Moreover, mitochondrial mass was determined, in parallel, using MitoTracker Deep-Red. Note that the ATP-high MCF7 cell subpopulation was enriched nearly 4-fold in CD44 cell surface expression, ~5.5-fold in ALDH activity, and nearly 3-fold in mitochondrial mass, when using a FACS gating cut-off of 5%. These findings are consistent with the enrichment of CSC activity. Data represent the mean fold increase over ATP-low 5% cells ±SD, *n* = 3. Unpaired *t* test, **p < 0.005, ***p < 0.0005. **H** ATP-high cells show increased capacity for 3D anchorage-independent growth. 3D anchorage-independent growth in MCF7 cells was assessed using the 3D mammosphere assay, after metabolic fractionation. Cells in 2D monolayers were first stained with BioTracker ATP-Red 1 and sorted by ATP content using the SONY SH800 flow cytometer. After cell counting, 5 × 10^3^ cells were seeded into poly-HEMA coated 6-well plates and counted after 5 days. Note that ATP-high MCF7 cells showed an increased capacity to undergo 3D anchorage-independent growth, relative to bulk cells and ATP-low MCF7 cells. ATP-high 5% (*n* = 6), Bulk 5% (*n* = 3), ATP-low 5% (*n* = 6). Data represent mean fold increase over ATP-low 5% cells ±SD. One-way ANOVA, Dunnett’s multiple comparisons test, ****p < 0.0001. Images of 3D mammospheres were acquired using the EVOS FL Auto2 microscope. The panels represent the three sorted cell populations. Representative Images are shown. A 4× objective was used. Scale bar = 1000 µm. **I** Other fluorescent probes for anti-oxidant capacity and pluripotency independently select for the ATP-high population. The effectiveness of BioTracker ATP-Red 1 was compared with several other fluorescent vital dyes for selecting the ATP-high cell population. MCF7 cell 2D monolayers were harvested with trypsin and lived-stained with a panel of five other fluorescent probes. Then, total ATP levels were determined using Cell-Titer-Glo, following flow cytometry. Note that the probes for anti-oxidant capacity (cystine-FITC and gamma-glutamyl-transpeptidase (GGT) activity) and pluripotency, all selected for the ATP-high subpopulation. The hypoxia probe also positively selected the ATP-high cell subpopulation. However, the senescence probe (beta-galactosidase activity) did not select for either the ATP-high or the ATP-low cell population. Data represent the mean fold increase over Probe-low 5% cells ± SEM, n = 3. Sidak’s multiple comparisons test, ns = not significant, *p < 0.05, ***p < 0.0005, ****p < 0.0001.

To test this “ATP hypothesis” further, we set out to identify and purify the energetically “fittest” cancer cells from within the total 2D cell population. For this purpose, we chose BioTracker ATP-Red 1, a fluorescent vital dye, to label ATP in living cancer cells (Fig. 2A).

BioTracker ATP-Red 1 is normally non-fluorescent, but becomes fluorescent when bound to ATP, but not any other related nucleotides or metabolites, including ADP [25]. Importantly, this ATP-biosensor exhibits a “turn-on” fluorescence-response toward ATP, with a near sixfold fluorescence enhancement [25]. Using fluorescence microscopy, ATP-Red 1 is predominantly detected within mitochondria [25], the major source of cellular ATP production (Fig. S7).

Therefore, we used ATP-Red 1 as a fluorescent probe to energetically fractionate the cancer cell population by flow cytometry, into ATP-high and ATP-low cell subpopulations, which were then subjected to phenotypic characterization. We tested various FACS gate cut-offs for cell selection and collection but focused mainly on 5 and 10%.

Figure 2A shows an example of bioenergetic fractionation with ATP-Red 1. First, we selected all live cells, in the singlet range. Then, MCF7 cells were subjected to metabolic fractionation by flow cytometry with ATP-Red 1, to isolate ATP-high (top 5%) and ATP-low (bottom 5%) cell subpopulations. The bulk (5%) population was also selected for comparison purposes. Importantly, based on mean signal intensity, we estimate that ATP-high MCF7 cells have approximately 15-fold higher levels of ATP, as compared with the ATP-low population; and 2-fold higher levels of ATP, as compared with the bulk cell population.

The selectivity of ATP-Red 1 was independently confirmed, by using Cell-Titer-Glo to measure ATP levels in cells, after flow cytometry. However, as Cell-Titer-Glo is a luciferase-based assay, it requires cell lysis to detect intracellular ATP levels and cannot be used for live-cell sorting or imaging. After cell counting, equal numbers of single cells were then used to evaluate their relative ATP content by luminescence, using the Varioskan^™^ LUX plate reader. Figure 2B shows that ATP-high MCF7 cells have a >15-fold increase in ATP levels, while bulk cells showed a ~7-fold increase ATP, all relative to the ATP-low cell population.

Next, we assessed cell proliferation, using a continuous real-time assay system. Figure 2C, D graphically shows the behavior of all three cell subpopulations (ATP-low 5%, Bulk 5%, and ATP-high 5%). Our results indicate that the ATP-high population is approximately twofold more proliferative, relative to the bulk cell population and approximately fivefold more proliferative, relative to the ATP-low population, after 120 h of monitoring. Therefore, we conclude that metabolic cell fractionation with an ATP-binding fluorescent dye reveals that high mitochondrial ATP levels are a key determinant of MCF7 cell proliferation. Similar results were obtained with three other breast cancer cell lines (Fig. S7).

### Metabolic analysis of 3D-mammospheres and ATP-high MCF7 cells

To better understand the metabolism underlying 3D-anchorage independent growth, we compared the intracellular ATP levels in MCF7 cells, cultured either as 2D monolayers or 3D mammospheres; the latter cell population is known to be highly enriched in CSCs. Interestingly, quantitative analysis of MCF7 cells derived from 3D mammospheres showed an ~2.3-fold increase in ATP levels, relative to 2D monolayer cells (Fig. 2E). Approximately, twofold increases in both the GSH/GSSG ratio and NADP/H levels were observed, as well (Fig. 2E); similar results were obtained with NAD/H. This is consistent with the idea that 3D anchorage-independent growth may also require increased anti-oxidant capacity.

Thus, we speculated that a subpopulation of 2D monolayer cells, with higher ATP levels, could have been self-selected for their ability to undergo 3D anchorage-independent growth. Under conditions of low attachment, >90% of MCF7 cells normally undergo anoikis, a specialized form of apoptotic cell death. Higher ATP levels would presumably allow CSCs to better resist the high stress of growth in suspension, caused by the absence of cell-substrate attachment. However, higher energy reserves might also confer resistance to multiple stressors, resulting in multi-drug resistance.

To test this hypothesis, we next subjected ATP-high and ATP-low MCF7 cells to metabolic analysis for NAD/H and two key anti-oxidants (namely, GSH and NADP/H). Remarkably, Fig. 2F shows that ATP-high cells contain >1.5-fold more NAD/H, >7.5-fold more NADP/H and >7-fold more reduced glutathione (GSH/GSSG ratio), all relative to ATP-low cells. Therefore, our results are consistent with the idea that ATP-high MCF7 cells are more energetic and, as consequence, they fortify their anti-oxidant capacity. High levels of antioxidants are known to be associated with drug resistance in cancer cells, possibly suggesting a multi-drug resistant phenotype. Therefore, we conclude that the ATP-high MCF7 subpopulation mimics the 3D metabolic phenotype.

### ATP-high MCF7 cells are energetically hyper-active, with increases in cancer stem cell markers, mitochondrial mass and 3D anchorage-independent growth

We examined “stemness” using two well-established CSC markers, namely CD44 and ALDH activity. Figure 2G shows that the ATP-high MCF7 cell subpopulation was enriched nearly 4-fold in CD44 cell surface expression and ~5.5-fold in ALDH-activity when using a FACS gating cut-off of 5%. Similar results were also obtained with MitoTracker-Deep-Red, a well-established marker of mitochondrial mass, which revealed a threefold increase in ATP-high MCF7 cells. We have previously shown that mitochondrial mass is a specific marker of stemness in CSCs [17, 26, 27].

We next employed the 3D-mammosphere assay to measure anchorage-independent growth, which is a functional read-out for CSC activity and CSC propagation. Interestingly, the ATP-high MCF7 cell subpopulation showed a ninefold increase in 3D spheroid formation, when using a FACS gating cut-off of 5% (Fig. 2H). These data directly support our hypothesis that ATP-high cells would be better able to undergo 3D anchorage-independent growth.

These findings are all consistent with the enrichment of CSC activity. Importantly, high ALDH activity is considered to be a biomarker of the epithelial-mesenchymal transition in CSCs, whereas CD44 is considered to be more of an epithelial CSC marker. So, both epithelial and mesenchymal CSCs are significantly enriched in the ATP-high cell subpopulation.

### Fluorescent vital probes for anti-oxidant capacity and pluripotency select for a population of ATP-high MCF7 cells

We also compared the effectiveness of the BioTracker ATP-Red 1 probe with several other fluorescent vital dyes for selecting the ATP-high cell population. For this purpose, MCF7 cell 2D monolayers were harvested with trypsin and lived-stained with a panel of five other fluorescent BioTracker probes for (i) anti-oxidant capacity (cystine uptake or gamma-glutamyl-transpeptidase (GGT) activity), (ii) pluripotency, (iii) hypoxia, and (iv) senescence (beta-galactosidase activity). Then, total ATP levels were determined using Cell-Titer-Glo, immediately following flow cytometry.

Figure 2 (panel I) shows the results of this analysis. Remarkably, the probes for anti-oxidant capacity (cystine uptake and GGT activity), as well as pluripotency, all selected for the ATP-high subpopulation of MCF7 cells. However, the BioTracker probe, which directly measures the uptake of cystine-FITC, was the most effective at selecting the ATP-high cell population but was not as effective as ATP-Red 1 (3-fold vs. ~20-fold). Interestingly, high anti-oxidant capacity is known to be strictly associated with stemness and the drug-resistance phenotype.

Surprisingly, the hypoxia probe also positively selected the ATP-high cell subpopulation. This may be due to the association between hypoxia and increased mitochondrial biogenesis.

However, the senescence probe (beta-galactosidase activity) did not select for either the ATP-high or the ATP-low cell population.

### ATP-high subpopulations of MCF7, T47D, MDA-MB-231, and MDA-MB-468 cells all overexpress ATP5F1C, are hyper-metabolic and show increased 3D anchorage-independent growth, as well as elevated cell cycle progression

**Fig. 3:**
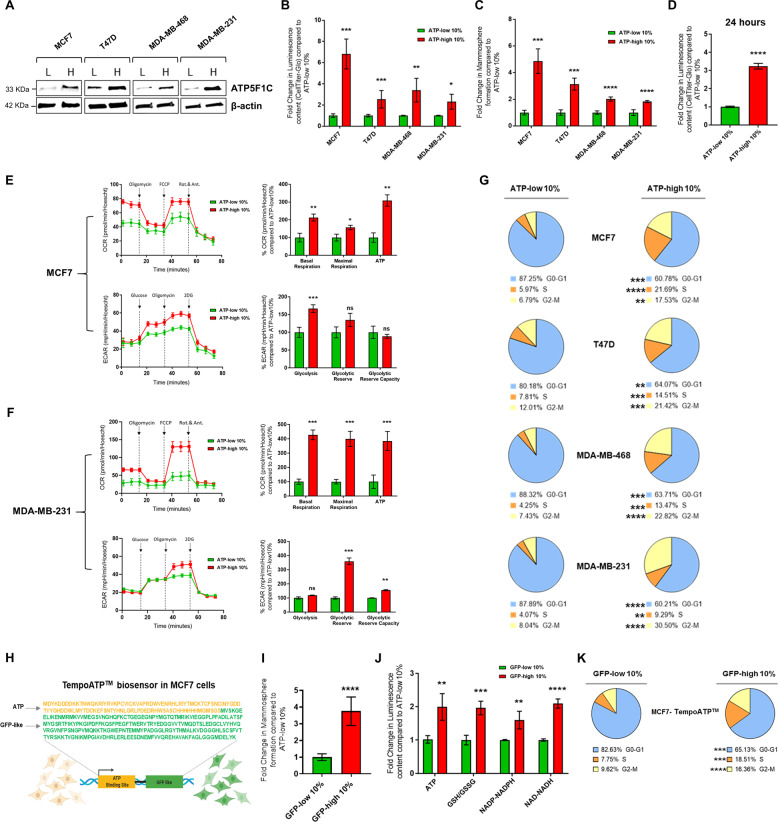
ATP-high subpopulations of MCF7, T47D, MDA-MB-231, and MDA-MB-468 cells all overexpress ATP5F1C, are hyper-metabolic, and show increased 3D anchorage-independent growth, as well as elevated cell cycle progression. **A** ATP5F1C is overexpressed in the ATP-high cell subpopulation. After metabolic fractionation, the ATP-high and ATP-low subpopulations were subjected to Western blot analysis with a mono-specific antibody probe that specifically recognizes ATP5F1C. Note that ATP5F1C is overexpressed in the ATP-high subpopulation in all four cell lines tested. **B** ATP levels after metabolic fractionation. The relative amount of ATP in the ATP-high and ATP-low cell subpopulations was independently validated using Cell-Titer-Glo. 2D monolayer cells were first stained with BioTracker ATP-Red 1 and sorted by ATP content, using a flow cytometer. After cell counting, equal numbers of single cells were then used to evaluate their relative luminescence content. In this series of experiments, we used a cut-off of 10% to define the ATP-high and ATP-low cell populations. Note that this metabolic fractionation scheme can be successfully applied to other breast cancer cell lines. Data represent the mean fold increase over ATP-low 10% cells ±SD, *n* = 3. Unpaired *t* test, *p < 0.05, **p < 0.005, ***p < 0.0005. **C** Mammosphere formation. Anchorage-independent growth was quantitated using the 3D mammosphere formation assay. In this series of experiments, we used a cut-off of 10% to define the ATP-high and ATP-low cell populations. Cells in 2D monolayers were first stained with BioTracker ATP-Red 1 and sorted by ATP content, using a flow cytometer. After cell counting, 5 × 10^3^ cells were seeded onto a poly-HEMA coated 6-well plate and counted after 5 days. Note that the ATP-high cell population of MCF7, T47D, MDA-MB-231, and MDA-MB-468 cells all showed an increased capacity for 3D anchorage-independent growth. Data represent the mean fold increase over ATP-low 10% cells ±SD, *n* = 3. Unpaired *t* test, ***p < 0.0005, ****p < 0.0001. **D** ATP levels remain high, even 24 h after plating. ATP levels in different MCF7 cell subpopulations were quantitated using Cell-Titer-Glo analysis. After cell counting, equal numbers of single cells were then used to evaluate their relative ATP content by luminescence using the Varioskan^™^ LUX plate reader, 24 h after plating. For these experiments, which required larger numbers of cells, we used a cut-off of 10% to define the ATP-high and ATP-low cell populations. Note that ATP-high cells showed a greater than threefold increase in ATP levels, as compared to ATP-low cells. Data represent the mean fold increase ±SD over ATP-low 10% cells, *n* = 3. Unpaired *t* test, ****p < 0.0001. **E** Metabolic flux analysis of ATP-high MCF7 cells. The OCR (oxygen consumption rate) and the ECAR (extracellular acidification rate) were determined using the Seahorse XFe96, via metabolic flux analysis. Note that the ATP-high MCF7 cell population shows an increase in both basal and maximal respiration, as well as mitochondrial ATP-production. The ATP-high MCF7 cell population also shows an increase in glycolysis. Cell populations were analyzed 24 h after plating. Data represent the % average fold increase ±SD over ATP-low 10% cells, *n* = 3. Unpaired *t* test, ns = not significant, *p < 0.05, **p < 0.005, ***p < 0.0005**. F** Metabolic flux analysis of ATP-high MDA-MB-231 cells. The OCR (oxygen consumption rate) and the ECAR (extracellular acidification rate) were determined using the Seahorse XFe96, via metabolic flux analysis. Note that the ATP-high MDA-MB-231 cell population shows an increase in both basal and maximal respiration, as well as mitochondrial ATP-production. The ATP-high MDA-MB-231 cell population also shows an increase in glycolytic reserve and glycolytic reserve capacity. Cell populations were analyzed 24 h after plating. Data represent the % average fold increase ±SD over ATP-low 10% cells, *n* = 3. Unpaired *t* test, ns = not significant, *p < 0.05, **p < 0.005, ***p < 0.0005. **G** Cell cycle progression. Note that the ATP-high cell subpopulation is hyper-proliferative, with increases in cell cycle progression, showing a strong shift away from the G0/G1-phase, toward the S-phase and the G2/M-phase. Quantitatively similar results were obtained with MCF7, T47D, MDA-MB-231, and MDA-MB-468 cell lines. In this series of experiments, we used a cut-off of 10% to define the ATP-high and ATP-low cell populations. The percentage of cells in the G0/G1, S, and G2/M phases of the cell cycle are reported. The results are expressed as the mean from three independent experiments, Unpaired *t* test, **p < 0.005, ***p < 0.0005, ****p < 0.0001. **H** Tempo-ATP: fluorescent-protein biosensor. Tempo-ATP is a fluorescent-protein biosensor for the detection of cytosolic ATP levels. This protein biosensor was stably expressed in MCF7 cells. The Tempo-ATP-biosensor allows for the measurement of ATP levels in a real-time fashion. As such, high GFP-fluorescent content is a reporter for high ATP content within the cells. The protein sequence of the ATP-binding portion of the biosensor is shown in yellow lettering, while the while GFP-like protein sequence is shown in green. Cells were sorted by GFP content, using flow cytometer (excitation = 517–519 nm; emission = 535 nm). **I** Tempo-ATP: mammosphere formation. Anchorage-independent growth was quantitated, using the 3D mammosphere formation assay. In this series of experiments, we used a cut-off of 10% to define the GFP-high and GFP-low cell populations. Cells in the monolayer were first sorted for GFP content, by flow cytometry. After cell counting, 5 × 10^3^ cells were seeded in poly-HEMA coated 6-well plates and counted after 5 days. Note that the number of 3D mammospheres formed was nearly fourfold increased in GFP-high MCF7 cells, as compared with GFP-low MCF7 cells, respectively. Data represent the mean fold increase over GFP-low 10% cells ±SD, *n* = 3. Unpaired *t* test, ****p < 0.0001. **J** Tempo-ATP: metabolic analysis. Metabolic profiling of key metabolites in GFP-high and GFP-low TempoATP-MCF7 cells. Luminescence levels were measured in GFP-high and GFP-low TempoATP-MCF7 cell subpopulations, using Promega kits (Cell-Titer-Glo, GSH/GSSG-Glo, NADP-NADPH-Glo, and NAD-NADH-Glo). Cells were sorted by GFP content, using flow cytometry. After cell counting, equal numbers of single cells were then used to evaluate their relative luminescence content. Note that GFP-high MCF7 cells showed a near 2-fold increase in ATP levels; a 2-fold increase in reduced glutathione levels; a ~1.5-fold increase in NADP-NADPH levels; and >2-fold increase in NAD-NADH levels, all relative to GFP-low cells. Data represent the mean fold increase over GFP-low 10% cells ±SD, *n* = 4. Unpaired *t* test, **p < 0.005, ***p < 0.0005, ****p < 0.0001. **K** Tempo-ATP: cell cycle progression. The GFP-high MCF7 cell subpopulation is hyper-proliferative, with increases in cell cycle progression, showing a strong shift away from the G0/G1-phase, towards the S-phase and the G2/M-phase. In this series of experiments, we used a cut-off of 10% to define the GFP-high and GFP-low cell populations. The percentage of cells in the G0/G1, S, and G2/M phases of the cell cycle are reported. The results are expressed as the mean from three independent experiments, Unpaired *t* test, *** *p* < 0.0005, ****p < 0.0001.

To examine the generality of these findings, we also prepared ATP-high and ATP-low cell subpopulations from three other human breast cancer cell lines, namely T47D, MDA-MB-231, and MDA-MB-468 cells. After metabolic fractionation, the ATP-high and ATP-low subpopulations were subjected to Western blot analysis with a mono-specific antibody probe that specifically recognizes ATP5F1C. Importantly, ATP5F1C is overexpressed in the ATP-high subpopulation, in all four cell lines tested (Fig. 3A).

Figure 3B illustrates that the ATP-high subpopulations of all these cell lines showed characteristic increases in ATP, as confirmed using the luciferase-based Cell-Titer-Glo assay, with a two to threefold increase in total ATP levels. This was also confirmed by directly imaging the ATP-high and ATP-low cell populations, by fluorescence microscopy (Figs. S8 and S9).

Strikingly similar results were also obtained with the 3D spheroid assay, indicative of an increase in CSC activity and propagation between 1.75- and 3-fold, depending on the cell line examined (Fig. 3C).

Interestingly, in MCF7 cells, the observed increases in ATP levels (Fig. 2A, F) were reduced to 3-fold by 24 h after plating the ATP-high cells as a 2D monolayer, indicating that this highly energetic phenotype is relatively transient, consistent with a more stem-like phenotype (Fig. 3D).

Next, we used metabolic flux analysis, with the Seahorse XFe96, to determine the energetic profiles of mitochondrial function and glycolytic rates (Fig. 3E). Interestingly, 24 h after cell attachment, ATP-high MCF7 cell monolayers showed a 2-fold increase in basal respiration, a 1.5-fold increase in maximal respiration and a 3-fold increase in mitochondrial ATP production. Similarly, ATP-high MCF7 monolayer cells also showed a 1.5-fold increase in their basal glycolytic rate. Therefore, ATP-high cells were clearly more bioenergetic than their ATP-low counterparts. Quantitatively similar results were also obtained with MDA-MB-231 cells (Fig. 3F).

We evaluated the proliferative capacity of the ATP-high cell subpopulation by measuring the status of cell cycle progression, using FACS analysis with propidium iodide to detect DNA content. Figure 3G directly shows that the ATP-high cell subpopulations were strikingly more proliferative than the ATP-low, with a shift from the G0/G1-phase to the S-phase and the G2/M-phase. More specifically, the G0/G1-phase was reduced from approximately 80–88% to 60–64%, while the S-phase was increased from 4–8% to 9–21%. Similarly, the G2/M-phase was increased, from 7–12% to 17–30%. Remarkably, these clear increases in cell cycle progression in the ATP-high cell subpopulation, relative to the ATP-low subpopulation, were observed in all four cell lines.

Importantly, quantitatively similar results were obtained with a second-independent ATP-biosensor, recombinantly expressed in MCF7 cells (Fig. 3H–K).

### ATP-high cells show features of multi-drug resistance: implications for understanding the relationship between mitochondrial ATP levels, drug resistance, and dormancy

**Fig. 4:**
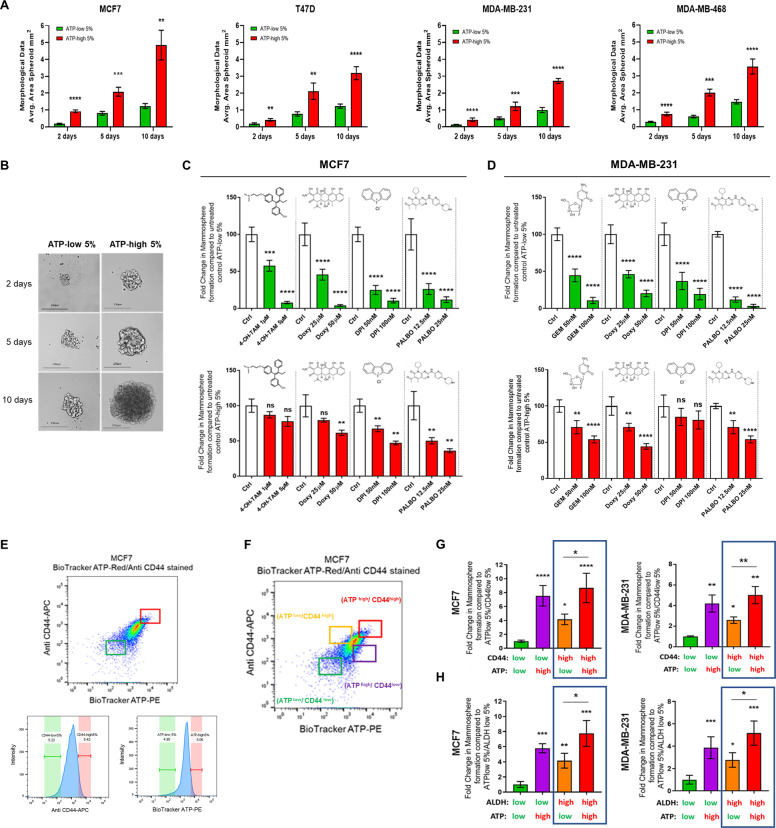
ATP-high cells show features of multi-drug resistance: Implications for understanding the relationship between mitochondrial ATP levels, drug resistance, and dormancy. **(A-B)** ATP-high cells form larger 3D spheroids. The average area of spheroids derived from MCF7, T47D, MDA-MB-231 and MDA-MB-468 cell lines was calculated, using ImageJ software. Note that ATP-high cells were able to generate much larger spheroids, as compared with ATP-low cells (**A**). Data represent the average fold increase ±SD over ATP-low cells in all subpanels, *n* = 3. Two-way ANOVA, Tukey corrected **p < 0.005, ***p < 0.0005, ****p < 0.0001. Images of ATP-low and ATP-high MCF7 cellular spheroids were acquired using an EVOS FL Auto2 microscope. Time points at 2, 5, and 10 days of growth are shown for the two sorted subpopulation cells. Representative images of MCF7 spheroid formation are shown (**B**). Note that ATP-high cells generated larger spheroids, as compared with ATP-low cells. For imaging, a 10× objective was used; a scale bar of 250 µm is shown. **C** ATP-high MCF7 cells are drug-resistant. We evaluated the differential sensitivity of ATP-high and ATP-low MCF7 cell subpopulations to four different classes of drugs, using the 3D mammosphere assay as a functional readout of drug resistance. Note that ATP-high MCF7 cells were clearly more resistant to treatment with Doxycycline (a mitoribosome inhibitor), Tamoxifen (an anti-estrogen), DPI (a mitochondrial OXPHOS inhibitor), and Palbociclib (a CDK4/6 inhibitor), consistent with a multi-drug resistance phenotype, all compared with the ATP-low 5% MCF7 cells. Data represent % average fold change ±SD over control 3D mammospheres in both panels, *n* = 3. One-way ANOVA, Dunnett’s multiple comparisons test, ns = not significant, **p < 0.005, ***p < 0.0005, ****p < 0.0001. **D** ATP-high MDA-MB-231 cells are drug-resistant. We evaluated the differential sensitivity of ATP-high and ATP-low MDA-MB-231 cell subpopulations to four different classes of drugs, using the 3D mammosphere assay as a functional readout of drug resistance. Note that ATP-high MDA-MB-231 cells were clearly more resistant to treatment with Doxycycline (a mitoribosome inhibitor), Gemcitabine (a DNA synthesis inhibitor), DPI (a mitochondrial OXPHOS inhibitor) and Palbociclib (a CDK4/6 inhibitor), consistent with a multi-drug resistance phenotype, all compared with the ATP-low 5% MDA-MB-231 cells. Data represent % average fold change ±SD over control 3D mammospheres in both panels, *n* = 3. One-way ANOVA, Dunnett’s multiple comparisons test, ns = not significant, **p < 0.005, ***p < 0.0005, ****p < 0.0001. **E** Comparing the effectiveness of BioTracker-ATP-Red 1, with other well-established markers of stemness. Representative flow cytometry profiles of BioTracker-ATP (PE-channel) and Anti-CD44 (APC-channel) in MCF7 cells, using the SONY SH 800 cell sorter. Unstained, BioTracker-ATP-stained, Anti-CD44-stained and BioTracker-ATP/Anti-CD44 double-labeled cell profiles are shown. These gates were used to determine the +ve populations of cells, based on the two different staining parameters. The labeling procedure was applied to both MCF7 and MDA-MB-231 cell lines. The “Green gate” detected cells with ATP-low and CD44-low content; the “Red gate” detected the cells with ATP-high and CD44-high content. The gates were also represented on the intensity histograms. An analogous experimental procedure was also conducted by employing single and double-labeling, with BioTracker-ATP (PE channel) and ALDH-activity (APC channel). **F** Defining four-cell subpopulations by FACS gating. A representative flow cytometry profile is shown, using the SONY SH 800 cell sorter. The “Green gate” represents cells with both ATP-low and CD44-low content; the “Orange gate” represents cells with ATP-low and CD44-high content; the “Purple gate” represents cells with ATP-high content and CD44-low content; the “Red gate” represents cells with both ATP-high and CD44-high content. An analogous strategy was used for double-labeling with BioTracker ATP (PE channel) and ALDH-activity (APC channel). **G** ATP vs. CD44 cell surface expression. 3D anchorage-independent growth was measured in the different cell subpopulations, as a functional read-out of stemness, using both MCF7 and MDA-MB-231 lines, after cell sorting. Briefly, 2D-monolayers were first co-stained with both BioTracker-ATP (PE channel) and Anti-CD44 (APC-channel) and subjected to flow cytometry, using the SONY SH800 cell sorter. After cell counting, 5 × 10^3^ cells were seeded in poly-HEMA coated 6-well plates and 3D-mammospheres were counted 5 days after plating. Interestingly, ATP-high cells showed an increased capacity to undergo 3D anchorage-independent growth, as compared with all the cell subpopulations. Most notably, ATP-hi/CD44-lo cells were better able to undergo 3D anchorage-independent growth, as compared with ATP-lo/CD44-hi cells and ATP-lo/CD44-lo cells. However, ATP-hi/CD44-hi cells showed the most 3D anchorage-independent growth. Data represent the average fold increase ±SD, relative to ATP-lo/CD44-lo cells, in both panels, *n* = 3. One-way ANOVA, Dunnett’s multiple comparisons test, *p < 0.05, ** *p* < 0.005, ****p < 0.0001. **H** ATP vs. ALDH activity. 3D anchorage-independent growth was measured in the different cell subpopulations, as a functional readout of stemness, using both MCF7 and MDA-MB-231 lines, after cell sorting. Briefly, 2D monolayers were first co-stained with both BioTracker-ATP (PE channel) and for ALDH-activity (APC-channel) and subjected to flow cytometry, using the SONY SH800 cell sorter. After cell counting, 5 × 10^3^ cells were seeded in poly-HEMA coated 6-well plates and 3D mammospheres were counted 5 days after plating. Interestingly, ATP-high cells showed an increased capacity to undergo 3D anchorage-independent growth, as compared with all the cell subpopulations. Most notably, ATP-hi/ALDH-lo cells were better able to undergo 3D anchorage-independent growth, as compared with ATP-lo/ALDH-hi cells and ATP-lo/ALDH-lo cells. However, ATP-hi/ALDH-hi cells showed the most 3D anchorage-independent growth. Data represent the average fold increase ±SD, relative to ATP-lo/CD44-lo cells, in both panels, *n* = 3. One-way ANOVA, Dunnett’s multiple comparisons test, *p < 0.05, **p < 0.005, ****p < 0.0001. In panel **G**, note that CD44-hi/ATP-hi cells showed more anchorage-independent growth than CD44-hi/ATP-lo cells. In panel **H**, note that ALDH-hi/ATP-hi cells showed more anchorage-independent growth than ALDH-hi/ATP-lo cells. These results imply that BioTracker-ATP can be used in combination with CSC markers, to separate CSCs into highly proliferative and less proliferative populations. This less proliferative CSC subpopulation may represent a more “dormant” CSC phenotype. In panels **A**–**H**, a cut-off of 5% was used to define the ATP-high and ATP-low cell populations.

To independently validate our results with the mammosphere assay, we used a second distinct spheroid assay to measure 3D growth, which depends instead on the self-aggregation of single cells to form 3D spheroids. After self-aggregation, the average area of the spheroids derived from MCF7, T47D, MDA-MB-231, and MDA-MB-468 cell lines was calculated, using ImageJ software. Time points at 2, 5, and 10 days of growth are shown for the two BioTracker ATP-Red 1 stained, sorted, subpopulation of cells. Importantly, ATP-high cells were able to generate much larger spheroids, as compared with ATP-low cells (Fig. 4A). Representative images of MCF7 spheroid formation are shown (Fig. 4B).

Next, we evaluated the differential sensitivity of ATP-high and ATP-low MCF7 cell subpopulations to four different classes of drugs, using the 3D-mammosphere assay as a functional readout of drug resistance (Fig. 4C).

In this assay system, we first studied Tamoxifen, which is an FDA-approved drug routinely used to clinically target ER(+) breast cancer cells, that often leads to Tamoxifen-resistance and treatment failure, resulting in tumor recurrence and distant metastasis. Interestingly, 3D-mammosphere formation by ATP-low MCF7 cells was remarkably sensitive to Tamoxifen treatment, resulting in a reduction by ~40% at 1 μM and by >90% at 5 μM. In contrast, 3D-mammosphere formation by ATP-high MCF7 cells was strikingly resistant to Tamoxifen, as 3D-mammosphere formation remained high at 5 μM, representing >80% of the vehicle-treated control levels. Thus, ATP-high MCF7 cells are clearly Tamoxifen-resistant. Validation of the multi-drug resistance phenotype was obtained by testing several other drugs, including Doxycycline (an inhibitor of mitochondrial protein translation), DPI (an OXPHOS inhibitor), and Palbociclib (a CDK4/6 inhibitor).

Quantitatively similar results were obtained using MDA-MB-231 cells (Fig. 4D), indicating that these ATP-high, triple-negative, breast cancer cells were also multi-drug resistant to Gemcitabine, Doxycycline, DPI, and Palbociclib.

### Comparing BioTracker-ATP-Red 1, with well-established markers of stemness, CD44, and ALDH-activity: implications for dissecting dormancy

In order to directly compare the effectiveness of BioTracker ATP-Red 1 with other CSC markers, we used a double-labeling strategy, which was applied to both MCF7 and MDA-MB-231 cells. More specifically, cells were double-labeled for CD44 and ATP, using different fluorescent channels for detection. In the case of CD44 and ATP, this leads to 4 experimental groups: CD44-hi/ATP-hi, CD44-hi/ATP-lo, CD44-lo/ATP-hi, and CD44-lo/ATP-lo. Optimization of the double-labeling assay, with specific FACS gating, is presented in Fig. 4E, F.

After cell sorting, the resulting four subpopulations were then subjected to the 3D-mammosphere assays, as a functional read-out of stemness. As shown in Fig. 4G, CD44-lo/ATP-lo cells showed the least anchorage-independent growth, as predicted. Therefore, CD44-lo/ATP-lo cells were chosen as the point for normalization. Remarkably, two cell populations showed the most 3D anchorage-independent growth: CD44-hi/ATP-hi and CD44-lo/ATP-hi. Therefore, high levels of ATP are the dominant determinant of stemness, as compared with CD44, in both MCF7 and MDA-MB-231 cells.

If we consider only the CD44-hi population, then double-labeling with ATP, allowed us to separate the CD44-hi population into two subpopulations, one with high capacity for propagation (CD44-hi/ATP-hi) and one with low capacity for propagation (CD44-hi/ATP-lo). Therefore, the CD44-hi/ATP-lo population clearly showed significantly less anchorage-independent growth and may represent a more “dormant” subpopulation of CD44(+) CSCs (Fig. S10).

Virtually identical results were also obtained by double-labeling with ADLH-activity and ATP (Fig. 4H). As such, this may be a powerful new approach, for sub-fractionating CSCs into a more “active” population and a more “dormant” population, using ATP as a secondary marker for dormancy. Perhaps, more importantly, these results also suggest that ATP levels may be a functional regulator of dormancy in CSCs.

### Investigating the role of mitochondrial ATP in cell migration, invasion, and spontaneous metastasis

We hypothesized that ATP may also be an energetic biomarker for the process of cancer cell metastasis. For this purpose, we focused our efforts on MDA-MB-231 cells, as they are a well-established model for the study of cell motility and metastasis, both in vitro and in vivo.

**Fig. 5:**
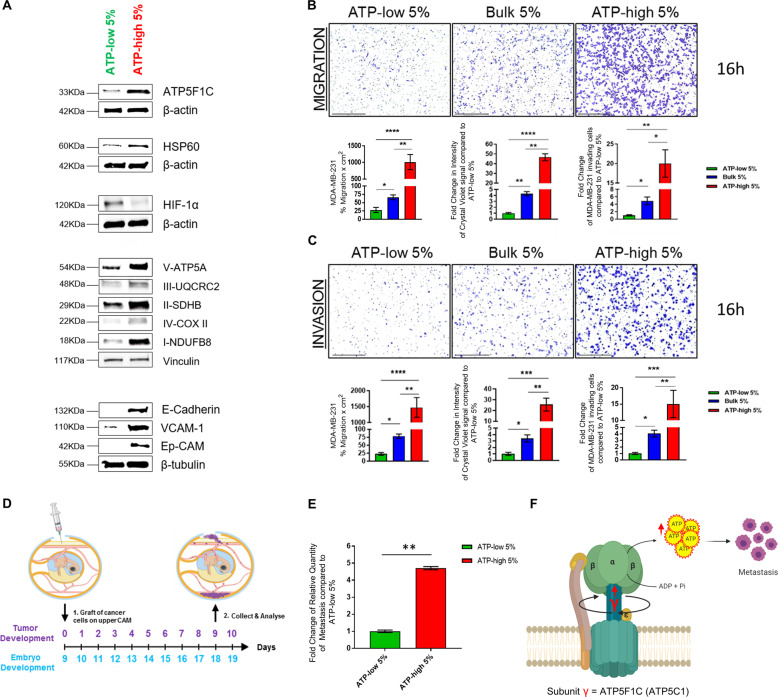
ATP-high MDA-MB-231 cells are enriched in CTC markers (EpCAM) and show increased migratory, invasive, and metastatic potential in vivo. **A** Western blot analysis reveals that mitochondrial markers and CTC markers are both upregulated in ATP-high MDA-MB-231 cells. Note that mitochondrial markers, from Complexes I to V (including ATP5F1C), as well as HSP60, were all over-expressed in ATP-high MDA-MB-231 cells, relative to ATP-low MDA-MB-231 cells. In addition, three known markers of CTCs and metastasis (VCAM-1, E-cadherin, and Ep-CAM) were all over-expressed in ATP-high MDA-MB-231 cells. In contrast, HIF-1α was highly expressed in ATP-low MDA-MB-231 cells. Beta-actin, Beta-tubulin, or Vinculin were used as controls for equal protein loading. **B**, **C** ATP-high MDA-MB-231 cells are migratory and invasive. MDA-MB-231 cells are a well-established model for the study of cell motility and metastasis, both in vitro and in vivo. Here, we evaluated the ability of ATP-high and ATP-low subpopulations of MDA-MB-231 cells to undergo cell migration and invasion, by employing a modified Boyden chamber assay, using Transwells. The bulk (5%) population was also selected for comparison purposes. Migration and invasion parameters were independently quantitated, using both crystal violet staining intensity and cell number. Representative images are shown. Scale bar = 500 μm. **B** Cell migration**:** Note that ATP-high MDA-MB-231 cells showed a 20–40-fold increase in their ability to undergo cell migration, relative to ATP-low cells. **C** Cell invasion: Similarly, ATP-high MDA-MB-231 cells showed a 15–60-fold increase in their ability to undergo invasion, relative to the ATP-low cell population. As expected bulk (5%) cells showed an intermediate phenotype, for both cell migration and invasion. One-way ANOVA, Dunnett’s multiple comparisons test, *p < 0.05, **p < 0.005, ***p < 0.0005, ****p < 0.0001. **D**, **E** ATP-high MDA-MB-231 cells represent the pro-metastatic cell subpopulation. We employed a well-established in vivo metastasis assay, involving the chorioallantoic membrane (CAM) assay in chicken eggs, to quantitatively measure spontaneous metastasis (**D**). Briefly, an inoculum of 30,000 cells (MDA-MB-231) was added onto the CAM of each egg (day E9) and then eggs were randomized into groups. On day E18, the lower CAM was collected to evaluate the number of metastatic cells, as analyzed by qPCR with specific primers for Human Alu sequences. Non-injected eggs were also evaluated in parallel, as a negative control for specificity. Note that ATP-high MDA-MB-231 cells were ~4.5-fold more metastatic than ATP-low cells, derived from the same cell line (**E**). As a consequence, it is likely that ATP-high MDA-MB-231 cells represent the pro-metastatic CSC subpopulation. *N* = 10 per group. Unpaired *t* test, **p < 0.005. **F** ATP5F1C is a potential driver of metastasis. Experimentally, we observe that high expression levels of ATP5F1C are correlated with high mitochondrial ATP production and are phenotypically associated with increased metastatic potential.

As a first step, we evaluated the possible co-expression of mitochondrial markers with markers of CTCs and metastasis, in ATP-high vs. ATP-low subpopulations. Remarkably, mitochondrial markers from Complexes I to V, including ATP5F1C, and HSP60 were all over-expressed in ATP-high MDA-MB-231 cells, relative to ATP-low MDA-MB-231 cells. In addition, three known markers of CTCs and metastasis (VCAM-1, E-cadherin, and Ep-CAM) were all overexpressed in ATP-high MDA-MB-231 cells (Fig. 5A). In contrast, HIF-1α levels were increased in ATP-low MDA-MB-231 cells.

Next, the ability of ATP-high and ATP-low subpopulations of MDA-MB-231 cells to undergo cell migration and invasion was evaluated by employing a modified Boyden chamber assay, using Transwells. The bulk (5%) population was also selected for comparison purposes.

Figure 5B shows the results of this analysis. Briefly, ATP-high MDA-MB-231 cells showed a 20-40-fold increase in their ability to undergo cell migration, relative to ATP-low cells. As expected bulk (5%) cells showed an intermediate phenotype. Similarly, ATP-high MDA-MB-231 cells showed a 15–60-fold increase in their ability to undergo invasion, relative to the ATP-low cell population (Fig. 5C). As such, ATP-high MDA-MB-231 cells may represent the pro-metastatic cell population in vivo.

To test this hypothesis directly, we employed a well-established in vivo metastasis assay, involving the CAM in chicken eggs, to quantitatively measure spontaneous metastasis (Fig. 5D).

Interestingly, Fig. 5E illustrates that ATP-high MDA-MB-231 cells were >4.5-fold more metastatic than ATP-low cells, derived from the same cell line. Therefore, ATP-high MDA-MB-231 cells likely represent the pro-metastatic CSC subpopulation (Fig. 5F). Importantly, they overexpress ATP5F1C (Fig. 5A) and functionally generate higher levels of mitochondrial ATP (Fig. 3F).

### shRNA-targeted knockdown of ATP5F1C protein expression inhibits ATP production, cell migration, and 3D anchorage-independent growth

Here, we identified ATP5F1C as a clinical biomarker of cancer metastasis (Fig. 1; Figs. S1–S6). In order to further validate the functional relevance of ATP5F1C as a potential therapeutic target, we next used an shRNA approach to down-regulate ATP5F1C expression in an inducible manner, by employing the Tet-On system, engineered into a single lentiviral vector.

**Fig. 6:**
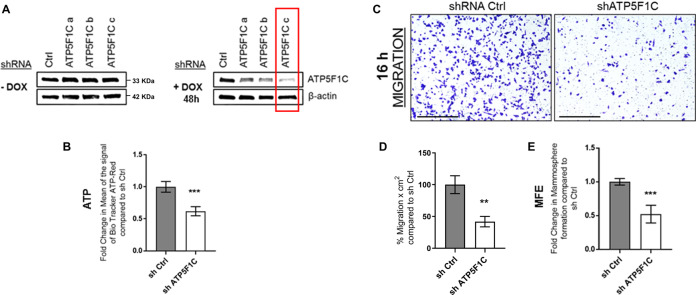
shRNA-targeted knockdown of ATP5F1C protein expression inhibits mitochondrial ATP production, cell migration, and 3D anchorage-independent growth. **A** Inducible knock-down of ATP5F1C. MDA-MB-231 cells were stably transduced with a lentiviral vector encoding an shRNA targeting ATP5F1C, in the Tet-On system; three different shRNA constructs were tested, namely a–c. MDA-MB-231 cells were also transduced with an shRNA-control vector in parallel. After 48 h of shRNA-induction by treatment with Doxycycline (10 μM), the levels of ATP5F1C were assessed by Western blot analysis. Note that the shRNA construct c showed largest inducible downregulation of ATP5F1C expression levels (indicated by the RED box). As a consequence, only cells transduced with construct c were used for further experiments, as compared with the shRNA control, both treated with Doxycycline. **B** Knockdown of ATP5F1C reduces ATP levels. Note that induced down-regulation of ATP5F1C reduces ATP levels by ~45%, relative to the shRNA control. Unpaired *t* test, **p < 0.005. **C**, **D** Knockdown of ATP5F1C inhibits cell migration: representative images (**C**) and quantitation (**D**). Note that induced down-regulation of ATP5F1C blocks cell migration by ~65%, relative to the shRNA control. MDA-MB-231 cells were cultured in presence of Doxycycline (10 μM) for 32 h and moved to Transwells for 16 h, all in presence of Doxycycline. Representative images are shown. Scale bar = 500 μm. Unpaired *t* test, **p < 0.005. **E** Knockdown of ATP5F1C inhibits 3D anchorage-independent growth. Note that induced downregulation of ATP5F1C blocks 3D mammosphere formation by ~50%, relative to the shRNA control. Unpaired *t* test, ***p < 0.0005. Therefore, ATP5F1C is a key regulator of mitochondrial ATP-production, cell migration, and 3D mammosphere growth.

Figure 6A shows that, using this approach, we were able to successfully down-regulate ATP5F1C expression in an inducible manner, by using low levels of Doxycycline. Importantly, by using this genetic approach to ablate ATP5F1C expression, we demonstrated that loss of ATP5F1C is indeed sufficient to phenotypically inhibit (i) ATP production, (ii) cell migration, and (iii) 3D anchorage-independent growth (Fig. 6B–E).

### Targeting ATP5F1C with Bedaquiline prevents ATP production, cell migration, 3D anchorage-independent growth, and metastasis in vivo

Bedaquiline is an FDA-approved antibiotic that is reserved for the treatment of multi-drug resistant tuberculosis (TB). Mechanistically, Bedaquiline inhibits the mycobacterial ATP-synthase. However, more recent studies have highlighted that Bedaquiline also specifically binds to the human mitochondrial ATP-synthase and potently inhibits its activity [28, 29]. Ultra-structurally, detailed cryo-EM studies have shown that the binding site of Bedaquiline is localized to the transmembrane F_0_ subunit of the yeast mitochondrial ATP-synthase [29]. The F_0_ subunit is directly connected to the F_1_ subunit, via the gamma-subunit (ATP5F1C), which functions as a rotating central stalk [29].

**Fig. 7:**
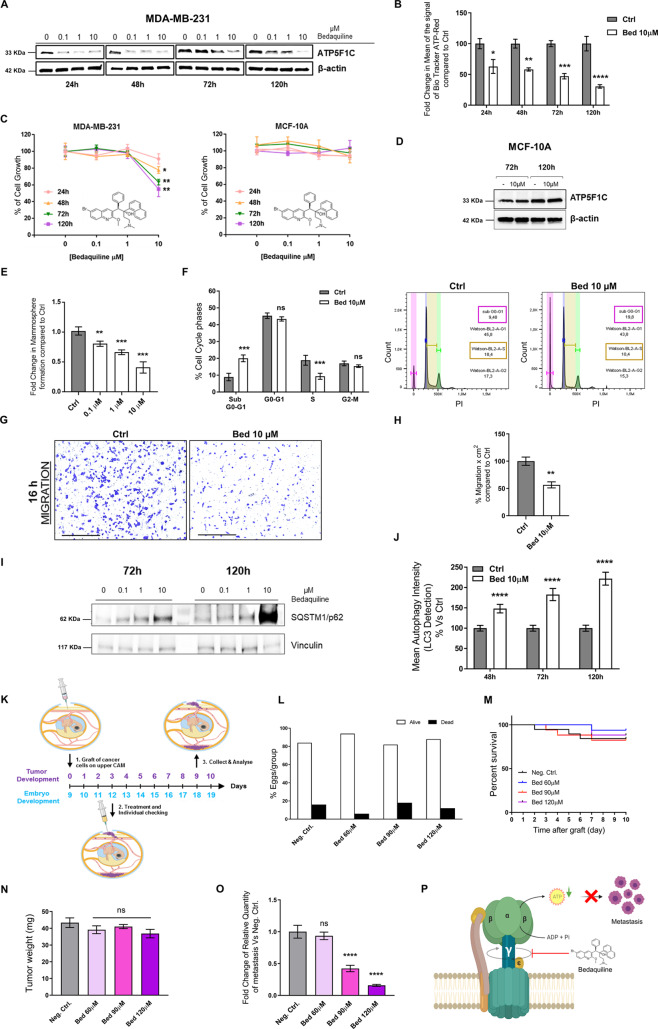
Targeting ATP5F1C with Bedaquiline prevents mitochondrial ATP production, cell migration, 3D anchorage-independent growth, and metastasis in vivo. **A** Bedaquiline downregulates the expression of ATP5F1C. MDA-MB-231 2D cell monolayers were treated with Bedaquiline (0, 0.1, 1, and 10 μM) and the expression of ATP5F1C was assessed by Western blot analysis at 4-time points (24, 48, 72, and 120 h of incubation). Note that the levels of ATP5F1C protein expression were reduced in a sustained way, especially at 10 μM Bedaquiline, relative to vehicle-alone (DMSO) controls. **B** Bedaquiline reduces mitochondrial ATP production. Note that Bedaquiline significantly inhibited ATP production in MDA-MB-231 cells, at a concentration of 10 μM, in a time-dependent manner, as assessed using Biotracker ATP-Red 1, to specifically detect ATP levels. Maximal inhibition of 75% was observed at 120 h of treatment. Two-way ANOVA, Sidak’s multiple comparisons test, *p < 0.05, ** *p* < 0.005, ***p < 0.0005, ****p < 0.0001. **C** Bedaquiline-induced ATP depletion reduces 2D monolayer cell growth in MDA-MB-231 cells, but not in MCF10A cells. Note that Bedaquiline effectively inhibits 2D growth in MDA-MB-231 cells, in a time-dependent manner, at a concentration of 10 μM. No effect was observed in MCF10A, which is a non-tumourigenic human mammary epithelial cell line. Unpaired *t* test, *p < 0.05, ** *p* < 0.005. **D** Bedaquiline does not reduce ATP5F1C expression in MCF-10A cells. MCF-10A cell monolayers were treated with Bedaquiline (10 μM) and the expression of ATP5F1C was assessed by Western blot analysis at the 2-time points: 72 and 120 h of incubation. Note that the levels of ATP5F1C protein expression were not reduced after 72 and 120 h of treatment with 10 μM Bedaquiline, relative to vehicle-alone (DMSO) controls. **E** ATP depletion inhibits 3D mammosphere formation, in a concentration-dependent manner. Note that Bedaquiline effectively blocks 3D mammosphere formation in MDA-MB-231 cells by ~65% at a concentration of 10 μM. One-way ANOVA, Dunnett’s multiple comparisons test, **p < 0.005, ***p < 0.0005. **F** ATP depletion inhibits DNA-synthesis and induces cell death: quantitation and representative FACS tracings. Note the twofold reduction of MDA-MB-231 cells in the S-phase and the concomitant twofold increase in the sub-G0–G1 population, after 120 h of treatment. Two-way ANOVA, Sidak’s multiple comparisons test, ns = not significant, ***p < 0.0005. Representative FACS tracings and Watson analysis of the cell cycle are shown. **G**, **H** ATP-depletion inhibits cell migration: Representative images (**G**) and quantitation (**H**). Note that Bedaquiline blocks MDA-MB-231 cell migration by ~50%. MDA-MB-231 cells were cultured in presence of Bedaquiline (10 μM) for 32 h and moved to Transwells for 16 h in the presence of Bedaquiline (10 μM). Representative images are shown. Scale bar = 500 μm. Unpaired *t* test, **p < 0.005. **I**–**J** ATP depletion using Bedaquiline induces autophagy in MDA-MB-231 cells. **I** MDA-MB-231 2D cell monolayers were treated with Bedaquiline (0, 0.1, 1, and 10 µM) and the expression of SQSTM1/p62 was assessed by Western blot analysis at 72 and 120 h. Note that the levels of SQSTM1/p62 protein expression were increased in a sustained way, especially at 10 µM Bedaquiline, relative to vehicle-alone (DMSO) controls. **J** Note that Bedaquiline increases the LC3B content in MDA-MB-231 cells. Autophagy Intensity was measured using the Autophagy LC3-antibody-based Kit for Guava® Muse® Cell Analyzer by Luminex. Cells were treated with Bedaquiline (10 µM) or vehicle alone, for 48, 72, and 120 h, and then subjected to analysis by flow cytometry. We observed an increase of LC3B content in Bedaquiline treated cells, based on the intensity of the signal. The analysis was conducted on 10,000 cells. Data represent the mean fold change ± SD over control (vehicle alone; DMSO) cells, *n* = 3. Two-way ANOVA, Sidak’s multiple comparisons test, **p < 0.005, ****p < 0.0001. **K** CAM assay for quantitating in vivo tumor growth, metastasis and toxicity. An inoculum of 1 × 10^6^ MDA-MB-231 cells was added onto the CAM of each egg (day E9) and then eggs were randomized into groups. On day E10, tumors were detectable, and they were then treated daily for 8 days with vehicle alone (1% DMSO in PBS) or Bedaquiline. After 8 days of drug administration, on day E18 all tumors were weighed, and the lower CAM was collected to evaluate the number of metastatic cells, as analyzed by qPCR with specific primers for Human Alu sequences. Before each drug administration, treatment tolerability was evaluated by scoring the number of live and dead chicken embryos. **L**, **M** Bedaquiline toxicity using the CAM assay: Live vs. Dead Assay (**L**) and % Survival (**M**). Note that Bedaquiline is non-toxic, relative to the vehicle-alone control. *N* ≥ 14 per group. **N** Tumor growth. Note that Bedaquiline has no significant effects on tumor growth in vivo. *N* ≥ 14 per group. One-way ANOVA, Dunnett’s multiple comparisons test, ns = not significant. **O** Metastasis. Note that Bedaquiline progressively inhibited spontaneous metastasis in vivo, in a dose-dependent manner, with a maximum inhibition of 84% at 120 μM. *N* ≥ 7 per group. One-way ANOVA, Dunnett’s multiple comparisons test, ns = not significant, ****p < 0.00005. **P** Schematic summary diagram. Bedaquiline, an FDA-approved drug, effectively inhibited mitochondrial ATP production and metastasis in vivo, by targeting the gamma subunit (ATP5F1C) of the mitochondrial ATP-synthase.

Therefore, we speculated that the binding of Bedaquiline to the transmembrane portion of mitochondrial ATP-synthase might induce the degradation of its key binding-partner, namely ATP5F1C. Figure 7A shows that, as predicted, the expression of ATP5F1C was indeed downregulated in response to Bedaquiline treatment, which was both time- and concentration-dependent. Notably, loss of ATP5F1C expression induced by Bedaquiline-treatment resulted in mitochondrial ATP depletion, by up to 75% (Fig. 7B). See also Supplemental Fig. S11.

More importantly, ATP depletion induced by Bedaquiline also inhibited the growth of MDA-MB-231 cells in 2D monolayers but did not affect the growth of MCF10A cells, a non-tumourigenic human breast epithelial cell line (Fig. 7C). Most notably, the expression of ATP5F1C was not affected in response to Bedaquiline treatment in MCF10A cells (Fig. 7D). Remarkably, ATP depletion was also not observed in MCF10A cells. See Supplemental Fig. S12.

Moreover, ATP depletion induced by Bedaquiline inhibited MDA-MB-231 cells from undergoing 3D anchorage-independent growth and cell migration; similarly, Bedaquiline treatment was sufficient to induce cell death in MDA-MB-231 cells, presumably by acting at the level of S-phase, to block cell cycle progression (Fig. 7E–H). See also Supplemental Fig. S13.

In addition, ATP depletion driven by Bedaquiline induced the expression of SQSMT1/p62, suggesting an autophagic-related mechanism of cell death (Fig. 7I). This was functionally confirmed by measuring LC3B levels in MDA-MB-231 cells treated with Bedaquiline (Fig. 7J). See also Supplemental Fig. S14.

Next, to test its in vivo activity, we used the well-established CAM assay, employing MDA-MB-231 cells, to measure Bedaquiline’s effects on tumor growth, spontaneous metastasis, and embryo toxicity (Fig. 7K). Importantly, Bedaquiline treatment was not toxic for the chick embryos, at any of the concentrations tested in vivo (Fig. 7L, M).

Surprisingly, Bedaquiline had no statistically significant effect on tumor growth (Fig. 7N). In striking contrast, Bedaquiline dose-dependently inhibited spontaneous metastasis, by up to 84% (Fig. 7O).

Therefore, we conclude that pharmacological targeting of the mitochondrial ATP-synthase with Bedaquiline can selectively prevent tumor cell metastasis, by inducing ATP-depletion, without driving systemic toxicity (Fig. 7P).

## Discussion

In this report, here we demonstrate that high mitochondrial ATP production is a key driver of “stemness” traits and proliferation in cancer cells. Our observations could explain the molecular basis of metabolic heterogeneity observed in the cancer cell population, as well as its relationship to phenotypic behaviors, such as (i) rapid cell cycle progression and (ii) anchorage-independent growth, which are both required for the metastatic dissemination of CSCs in vivo.

### ATP-related genes as biomarkers of tumor recurrence and metastasis: focus on ATP5F1C

Recently, we presented evidence that treatment with a panel of distinct anti-mitochondrial therapeutics, that target the mitochondrial ribosome, is sufficient to potently inhibit cancer cell metastasis, using an in vivo xenograft model [23, 24]. These results functionally imply that high ATP levels are critical for the process of CSC metastasis.

These findings are consistent with our current observations that ATP-high cancer cells are hyper-proliferative, stem-like, anchorage-independent, with increases in anti-oxidant capacity and intrinsic multi-drug resistance. Therefore, we speculate that the ATP-high CSCs that we have isolated here are likely to be responsible for tumor recurrence and metastasis in vivo.

This assertion is consistent with our bioinformatic analysis showing that ATP-related genes are closely associated with stemness, proliferation, and metastasis, especially ATP5F1C, which encodes the gamma subunit of the catalytic core of the mitochondrial ATP synthase. Moreover, we presented evidence that ATP5F1C is a prognostic biomarker of tumor recurrence and distant metastasis, as well as a marker of treatment failure in ER(+) patients undergoing Tamoxifen therapy.

In accordance with this idea, here we observed that ATP-high MDA-MB-231 cells showed dramatic increases in their capacity to undergo both cell migration and invasion in vitro, as well as spontaneous metastasis in vivo. These findings provide independent evidence for the critical role of mitochondrial ATP in the process of metastatic dissemination.

### Targeting ATP5F1C with Bedaquiline, an FDA-approved drug, prevents aggressive cancer cell behaviors, including spontaneous metastasis

Bedaquiline (a.k.a., Sirturo) is an FDA-approved antibiotic, that is clinically used for the treatment of drug-resistant TB (DR-TB). Originally, it was thought that Bedaquiline only affected the mycobacterial ATP-synthase, but more recent studies have indicated that Bedaquiline also potently inhibits the yeast and human mitochondrial ATP-synthase [28, 29].

High-resolution cryo-EM studies have shown that Bedaquiline binds directly to the c-ring of the six transmembrane proteins (ATP5G1/G2/G3), that form the F_0_ subunit of the mitochondrial ATP synthase [29]. In turn, the c-ring of the F_0_ subunit is directly connected to the F_1_ subunit, via the gamma-subunit (ATP5F1C). The gamma-subunit (ATP5F1C), which forms the rotary shaft of the mitochondrial ATP-synthase, is critically involved in torque transmission, ultimately providing the necessary mechano-chemical energy for ATP-synthesis [30–32].

As Bedaquiline-binding significantly alters the 3D conformation of the c-ring, we speculated that this conformational change would induce the degradation of ATP5F1C. In accordance with this hypothesis, we observed that Bedaquiline induced the down-regulation of ATP5F1C protein expression, with concomitant mitochondrial ATP depletion, in a time- and concentration-dependent manner. Furthermore, ATP depletion induced by Bedaquiline treatment was indeed sufficient to effectively block spontaneous metastasis in vivo, without significant toxicity in non-tumourigenic human cells (MCF10A) in vitro or chicken embryos in vivo.

As a consequence, we conclude that the gamma-subunit of the mitochondrial ATP-synthase (ATP5F1C) is a new therapeutic target, for mitigating aggressive cancer cell behaviors, including spontaneous metastasis.

## Supplementary information

Supplementary Info Final 090421

Supplemental Figure S1

Supplemental Figure S2

Supplemental Figure S3

Supplemental Figure S4

Supplemental Figure S5

Supplemental Figure S6

Supplemental Figure S7

Supplemental Figure S8

Supplemental Figure S9

Supplemental Figure S10

Supplemental Figure S11

Supplemental Figure S12

Supplemental Figure S13

Supplemental Figure S14
