# Supplementary material for: Bedaquiline, an FDA-approved drug, inhibits mitochondrial ATP production and metastasis in vivo, by targeting the gamma subunit (ATP5F1C) of the ATP synthase
Source: Cell Death Differ. 2021 May 13;28(9):2797–817. doi: 10.1038/s41418-021-00788-x (PMC8408289; doi:10.1038/s41418-021-00788-x)
Supplement: Supplementary file 1 — Supplementary Info Final 090421 [file 41418_2021_788_MOESM1_ESM.docx]

**Supplementary Information:**

**Bedaquiline, an FDA-approved drug, Inhibits Mitochondrial ATP Production and Metastasis *In Vivo*, by Targeting the Gamma Subunit (ATP5F1C) of the ATP Synthase**

Marco Fiorillo ^1,2^, Cristian Scatena ^3,4^, Antonio Giuseppe Naccarato ^3,4^, Federica Sotgia ^1^ and Michael P. Lisanti ^1^

^1^ Translational Medicine, School of Science, Engineering and Environment (SEE), University of Salford, Greater Manchester, United Kingdom

^2^ The Department of Pharmacy, Health and Nutritional Sciences, The University of Calabria, Cosenza, Italy

^3^ Division of Pathology, Department of Translational Research and New Technologies in Medicine and Surgery, University of Pisa, 56126 Pisa, Italy

^4^ Department of Laboratory Medicine, Pisa University Hospital, Anatomia Patologica 1 Universitaria, 56126 Pisa, Italy

**Supplemental Bioinformatic Information:**

As a first step, we re-analyzed GEO transcriptional profiling DataSets, comparing 2D-growth, 3D-growth and the *in vivo* tumour growth of MDA-MB-231 cells, a triple-negative breast cancer (TNBC) cell line ^1^. HeatMaps were generated, highlighting that ATP-related genes were transcriptionally upregulated under both 3D growth conditions (anchorage-independent and *in vivo* tumours), all relative to 2D-adherent growth (**Fig. S1A**).

Moreover, we examined the transcriptional expression of ATP-related genes (OXPHOS and ATP-related transporters), in two distinct GEO DataSets related to human breast cancer metastasis ^2, 3^ (**Fig. S1B, C**). Intersection of these two GEO DataSets resulted in a five-member ATP-related metastasis gene-signature, consisting of ABCA2, ATP5F1C, COX20, NDUFA2 and UQCRB (**Fig. S1D, E**). Most notably, ATP5F1C (also known as ATP5C1) encodes the gamma-subunit of the soluble F1-catalytic core of the mitochondrial ATP synthase, complex V (**Fig. S1F**).

Remarkably, in samples derived from patients with metastatic breast cancer, ATP5F1C transcriptional expression is positively correlated with the co-expression of: i) five metastatic marker genes (EPCAM, MKI67, RRP1B, VCAM1, CXCR4), ii) four cell cycle regulatory genes (CDK1, CDK2, CDK4, CDK6) and iii) eleven CSC marker genes (CDH1, ALDH2, ALDH1BA1, ALDH9A1, SOX2, VIM, CDH2, ALDH7A1, ALDH1B1, CD44, ALDH3B2, listed in rank order of statistical significance) (**Fig. S2**). Interestingly, ATP5F1C transcriptional expression is also positively correlated with the co-expression of mitochondrial complexes I-V, mt-DNA encoded transcripts and three other members of the five-member metastasis gene-signature, namely UQCRB, COX20 and NDUFA2 (**Fig. S1E and S2**).

Similarly, the expression of two members of this metastasis gene signature, namely ATP5F1C and UQCRB, have been functionally correlated with maximal oxygen uptake (V0_2max_) and a high percentage of type 1 fibers (mitochondrial-rich) in human skeletal muscle tissues ^4^. The expression of ATP5F1C in skeletal muscle is also increased significantly after exercise training, reflecting increased muscle fitness in patients; conversely, ATP5F1C levels decreased with advanced age ^4^ and were reduced in progeria syndrome patients ^5^. These results are highly suggestive that high ATP5F1C expression is a biomarker of increased mitochondrial ATP production, at the cellular level.

Using Kaplan-Meier (K-M) analysis, we determined that ATP5F1C is a prognostic biomarker for distant metastasis and tumour recurrence, especially in ER(+) patients that are lymph node negative at diagnosis and were treated with Tamoxifen (HR(RFS)=2.77; P=3.4E-06; N=471) (**Fig. S3**). Similarly, ATP5F1C has prognostic value in other epithelial cancers, such as ovarian and lung, among others (**Fig. S4**).

In addition, we examined if ATP-related genes and OXPHOS genes might be transcriptional biomarkers of breast cancer circulating tumour cells (CTCs) in patients, using existing GEO DataSets ^6^. The resulting HeatMaps are shown in **Supplemental Figure S5**. Overall, our analysis suggests that high ATP content in CTCs may be useful as a biomarker, to identify and track CTCs in whole blood, thereby potentially improving cancer diagnosis and preventing metastatic spread.

Finally, we re-interrogated existing proteomic profiling data, comparing 2D-monolayers with 3D-mammospheres, in two distinct ER(+) breast cancer cell lines, namely MCF7 and T47D. Overall, from 1,519 common proteins in both cell lines, 21 ATP-related proteins were found to be up-regulated in both data sets, in 3D-mammospheres (**Fig. S1G**). Out of these 21 ATP-related proteins, 7 subunits of the mitochondrial ATP-synthase were increased, including ATP5F1B, ATP5F1C, ATP5IF1, ATP5MG, ATP5PB, ATP5PD and ATP5PO.

Taken together, this bioinformatic analysis is consistent with the hypothesis that increased mitochondrial ATP synthesis could be a key driver of 3D anchorage-independent growth and metastasis.

**Supplemental Figures - Legends**

**Supplemental Figure S1.** **ATP-related genes are biomarkers of 3D growth and metastasis: Importance of ATP5F1C, the gamma-subunit of the mitochondrial ATP-synthase.**

**(A) ATP-related genes as biomarkers for 3D growth.** Here, we generated a HeatMap comparing the transcriptional profiles of ATP-related genes (OXPHOS and ATP-related transporters), using the GSE36953 GEO DataSet, previously deposited in the NCBI database. Total RNA was prepared from MDA-MB-231 cells, a TNBC cell line, under three different growth conditions: 2D-adherent growth, 3D-anchorage-independent growth and *in vivo* tumour growth. Analysis was performed with the Affymetrix Human Genome U133 Plus 2.0 Array. The HeatMap was generated with QIAGEN OmicSoft Suite Software. A -4<Log FC>+4 HeatMap scale bar is shown. Note that ATP-related genes were transcriptionally upregulated under both 3D growth conditions (anchorage-independent and *in vivo* tumours), all relative to 2D-adherent growth.

**(B-C)** **ATP-related genes as biomarkers of metastasis.** Volcano plots for the GSE2034 and GSE59000 GEO DataSets are shown. GSE2034 compares Breast Cancer Metastasis Vs No Breast Cancer Metastasis. GSE59000 compares Breast Cancer Metastasis Vs Breast Cancer Primary Tumour. Volcano plots were produced by examining the annotations present in OncoLand Metastatic Cancer (QIAGEN OmicSoft Suite) and by performing functional “core analyses” using Ingenuity Pathway Analysis Software (IPA; QIAGEN), on genes annotated with an uncorrected p-value cut off <0.05. Note that the transcriptional profiles of ATP-related genes (OXPHOS and ATP-related transporters), were increased and specifically associated with metastasis, in both GEO DataSets.

**(D)** **Five ATP-related genes (ABCA2, ATP5F1C, COX20, NDUFA2, UQCRB), as common biomarkers of metastasis.** A Venn diagram intersecting the two breast cancer metastasis GEO DataSets (GSE2034 and GSE59000) is shown. Intersection of the two GEO DataSets was performed, essentially as described in panels B & C, using IPA Software. Note that five ATP-related genes (ABCA2, ATP5F1C, COX20, NDUFA2, UQCRB) were highly upregulated in both metastasis GEO DataSets.

**(E)** **ATP5F1C gene expression correlates with the co-expression of other ATP-related genes in metastatic samples derived from N=146 breast cancer patients.**  Data with UQCRB, COX20, NDUFA2 and ABCA2 are shown here (primary data were extracted from The Metastatic Breast Cancer Project (Provisional, February 2020)). For further analysis of the co-expression of genes correlated with ATP5F1C, please also see **Supplemental Figure S2**.

**(F) Model of the mitochondrial ATP-synthase (Complex V).** Note that ATP5F1C is the gamma-subunit of the nanoscale rotary motor that produces ATP. ATP5F1C functions in the transmission of torque, providing the necessary mechano-chemical energy for generating ATP.

**(G)** **ATP-related protein biomarkers associated with 3D growth.** The proteomic profiles of two ER(+) breast cancer cell lines (MCF7 and T47D) were first compared, under 2D-adherent and 3D-anchorage-independent growth conditions. Then, these two data sets were intersected to create a Venn diagram. Commonly shared ATP-related gene products (OXPHOS and ATP-related transporters) are enumerated below the Venn diagram. Note that, from 1,519 common proteins, 21 ATP-related proteins were found to be upregulated in both data sets. Proteomic data sets were interrogated by performing functional “core analyses”, using Ingenuity Pathway Analysis Software (IPA; QIAGEN).

**Supplemental Figure S2. ATP5F1C gene expression positively correlates with the co-expression of markers of mitochondria, stemness, cell cycle progression and metastasis.**

Gene co-expression profiles were extracted from The Metastatic Breast Cancer Project Provisional (2020), using cBioPortal (cbioportal.corg; [Cerami et al., 2012](http://cancerdiscovery.aacrjournals.org/content/2/5/401.abstract) & [Gao et al., 2013](http://www.ncbi.nlm.nih.gov/pubmed/23550210)). mRNA expression profiling (RNA Seq V2 RSEM) was carried out via RNA-sequencing of tissue samples derived from N=146 patients with metastatic breast cancer. In panels **(A-L),** we summarize the statistical analysis correlating ATP5F1C gene expression, with the co-expression of other genes. Note that we report the positive correlations (in green), with a p-value <0.05.

**(A-H)** ATP5F1C gene expression positively correlates with the co-expression of mitochondrial complexes I-V, mt-DNA and other ATP-related genes/transporters.

**(I)** ATP5F1C gene expression positively correlates with the co-expression of three other metastasis-associated mitochondrial genes, UQCRB, COX20 and NDUFA2, but not ABCA2, an ABC transporter.

**(J)** ATP5F1C expression is positively correlated with the co-expression of breast CSC marker genes (CDH1, ALDH2, SOX2, VIM, CD44).

**(K)** ATP5F1C expression is positively correlated with the co-expression of CTC and metastatic marker genes (EPCAM, MKI67, RRP1B, VCAM1, CXCR4).

**(L)** ATP5F1C expression is positively correlated with the co-expression of cell cycle regulatory genes (CDK1, CDK2, CDK4, CDK6).

**Supplemental Figure S3. ATP5F1C gene expression is a transcriptional biomarker of tumour recurrence, distant metastasis and Tamoxifen-resistance.**

**(A-C)** Here, using Kaplan-Meier (KM) analysis, we determined that ATP5F1C is a prognostic biomarker for tumour recurrence and distant metastasis, especially in ER(+) patients that are lymph node (LN) negative at diagnosis and were treated with Tamoxifen (HR(RFS)=2.77; P=3.4E-06; N=471). To perform K-M analysis on ATP5F1C, we used an open-access online survival analysis tool to interrogate publicly-available microarray data from breast cancer patients. This approach allowed us to directly perform *in silico* validation of ATP5F1C as a marker of tumour recurrence **(A,C)** (**RFS,** replapse-free survival) and distant metastasis **(B)** (**DMFS,** distant metastasis-free survival).

**Supplemental Figure S4. ATP5F1C gene expression is a transcriptional biomarker of poor overall survival (OS) in ovarian carcinoma (serous-type) and lung adenocarcinoma.**

Here, using Kaplan-Meier (KM) analysis, we determined that ATP5F1C is a prognostic biomarker for overall survival in ovarian **(A)** and lung cancer **(B)** patients. To perform K-M analysis on ATP5F1C, we used an open-access online survival analysis tool to interrogate publicly-available microarray data from these populations of cancer patients. This approach allowed us to directly perform in silico validation of ATP5F1C as a marker of overall survival (OS).

**(A) Ovarian Carcinoma (serous-type).** Only patients with optimal debulking were selected for analysis, including stages 2-4. Biased array data were excluded. Data from N=554 patients were analysed. (HR=1.67; P=4e-05).

**(B) Lung Adenocarcinoma.** Only patients with negative surgical margins were selected for analysis. Biased array data were excluded. Data from N=204 patients were analysed. (HR=4.19; P=0.00065).

**Supplemental Figure S5. ATP-related genes and OXPHOS genes as transcriptional biomarkers of breast cancer circulating tumour cells (CTCs).**

Genes expression profiling data was extracted from GSE55470, accessible from GEO DataSets in the NCBI database (*https://www.ncbi.nlm.nih.gov/geo/query/acc.cgi? acc = GSE55470*). This study by Fina et. al., provides a reliable assay, that allowed us to obtain valuable information about the metabolic features of CTCs. Briefly, gene expression profiling was carried using CTCs isolated from the blood of advanced breast cancer patients. Moreover, as a positive control, MCF7 and MDA-MB-468 breast cancer cells were spiked into 5 mL of the whole blood isolated from healthy donors and captured using the AdnaTest EMT-1/StemCell Select kit (Qiagen). Total RNA was isolated and processed onto the Illumina Whole-Genome DASL HT platform. According to the characteristics of the study, data normalization was not applicable. In our bioinformatics analysis, we constructed HeatMaps of the transcriptional profiles of **(A)** ATP-related genes/transporters and **(B)** OXPHOS genes, as shown. Positive control samples were generated by spiking whole blood from healthy donors, with 50 cells each of either MCF7 or MDA-MB-468 cells. Samples from 5 advanced breast cancer patients, where the exact number of CTCs were known, are also shown, as indicated (containing 200, 7, 13, 13 and 19 CTCs, respectively). These samples were all compared with healthy donor control blood (N=5), as negative controls. Our analysis suggests that high ATP content in CTCs may be useful as a biomarker, to identify and track CTCs in whole blood, thereby improving cancer diagnosis and preventing metastatic spread. The HeatMap scale bar is as shown: -4<Log FC>+4.

**Supplemental Figure S6. Comparative analysis of ATP5F1C protein expression in normal breast tissue, DCIS, primary invasive breast carcinomas, and metastatic lesions.**

**(A) ATP5F1C protein expression is low or absent in normal breast tissue but is elevated in primary invasive breast carcinomas and ductal carcinoma *in situ* (DCIS).** Formalin-fixed paraffin-embedded (FFPE) tissue sections of normal breast tissue (Upper), normal breast tissue with adjacent invasive breast carcinoma (Middle), and DCIS (Lower), from the same patient with luminal breast cancer, were subjected to immuno-histochemical staining with mono-specific antibodies that recognize ATP5F1C. Representative examples are shown. Note that ATP5F1C expression is low or absent in normal tissue and increased substantially in adjacent invasive breast carcinoma and/or DCIS. Note that the tumour stromal tissue remains largely unstained. Images were acquired with a 20X objective. Scale bar = 100 µm.

**(B) High ATP5F1C protein expression is observed in diverse metastatic lesions.** Formalin-fixed paraffin-embedded (FFPE) tissue sections of metastatic lesions excised from the lymph-nodes (LNs; Upper), the brain (Middle) and bone marrow (Lower) of three different HER2(+) breast cancer patients were subjected to immuno-histochemical staining with mono-specific antibodies that recognize ATP5F1C. Representative examples are shown. Note that ATP5F1C is highly expressed in these metastatic lesions. Images were acquired with a 20X objective. Scale bar = 100 µm.

**Supplemental Figure S7. Using BioTracker ATP-Red 1 to obtain ATP-high and ATP-low sub-populations of cancer cells: ATP-high cells undergo hyper-proliferation.**

**(A) Detecting Mitochondrial ATP in living cells.** BioTracker ATP-Red 1 allows for the dynamic detection and visualization of mitochondrial ATP in living cells and tissues. BioTracker ATP-Red 1 is a vital dye that is only fluorescent when bound to ATP but does not recognize ADP or other nutrients. This ATP-biosensor exhibits a “turn-on” fluorescence-response toward ATP, with a near 6-fold fluorescence enhancement.

**(B) ATP-high MDA-MB-231 cells are hyper-proliferative**. Cell proliferation was assessed using the xCELLigence, a continuous real-time assay system. Cell tracings and slope analysis are shown.

**(C) ATP-high MDA-MB-468 cells are hyper-proliferative.** See panel B for details.

**(D) ATP-high T47D cells are hyper-proliferative.** See panel B for details.

For panels **(B-D),** the Unpaired t-test was used, **p* < 0.01.

**Supplemental Figure S8. Fluorescence Imaging of ATP-high and ATP-low Sub-populations of ER(+) Breast Cancer Cell Lines: MCF7 and T47D.**

ATP-high and ATP-low sub-populations of MCF7 and T47D cells were isolated by flow cytometry and equal numbers of cells were plated on a 2D surface. After 24 hours of culture, the cell populations were stained with two vital dyes: Hoescht 33342 (for DNA) and BioTracker ATP-Red 1 (for ATP). Note the differences in ATP-based fluorescence intensity in the ATP-high and ATP-low cell populations. Scale Bar = 200 μm.

**Supplemental Figure S9. Fluorescence Imaging of ATP-high and ATP-low Sub-populations of ER(-) Breast Cancer Cell Lines: MDA-MB-468 and MDA-MB-231.**

ATP-high and ATP-low sub-populations of MDA-MB-468 and MDA-MB-231 cells were isolated by flow cytometry and equal numbers of cells were plated on a 2D surface. After 24 hours of culture, the cell populations were stained with two vital dyes: Hoescht 33342 (for DNA) and BioTracker ATP-Red 1 (for ATP). Note the differences in ATP-based fluorescence intensity in the ATP-high and ATP-low cell populations. Scale Bar = 200 μm.

**Supplemental Figure S10. Isolating “high-proliferation” and “low-proliferation” sub-populations of CSCs, using a double-labelling strategy: Separating activated and dormant fractions of CSCs.**

In a double-labelling strategy, here we combined ATP with the CSC marker CD44, to fractionate the CSC population into two sub-populations. More specifically, CD44-hi/ATP-hi cells showed twice the level of anchorage-independent growth, as compared to CD44-hi/ATP-lo cells. Therefore, CD44-hi/ATP-lo cells may represent a more dormant CSC population. These results suggest that ATP levels may be a functional regulator of dormancy in CSCs. Quantitatively similar results were obtained, by combining ATP with another CSC marker, namely ALDH-activity.

**Supplemental Figure S11. Bedaquiline differs from oligomycin in targeting the ATP5F1C protein and reducing ATP content levels.**

**(A-C) Cell growth analysis after Oligomycin treatment.** Proliferation was assessed using the xCELLigence^®^ RTCA DP instrument. 1 x 10^4^ cells were seeded (in common media) in RTCA DP E-Plates for real-time growth analysis. After 24h post-seeding, the cells were treated with 1, 2.5, 5, 7.5 µM of Oligomycin; vehicle alone (DMSO) was run as negative control. The results indicate that Oligomycin reduces MDA-MB-231 growth (Cell Index) in a concentration-dependent manner over 120 hours. Data represent the mean ± SEM, n=4.

**(B-C) Sulforhodamine B (SRB) assay in MDA-MB-231 and MCF-10A cells.** To assess cell viability, we also used the sulforhodamine B (SRB) end-point assay. Briefly, 5 × 10^3^ cells/well were seeded in 96-well plates and cultured overnight to allow the cells to attach. Then, cells were treated with Oligomycin (1, 2.5, 5 and 7.5 µM), for 120h. At the end of the treatment, cell monolayers were fixed with chilled 10% (wt/vol) trichloroacetic acid for 15 min at 4°C. Plates were then washed with PBS and allowed to dry. Next, cells were stained with 0.04% (wt/vol) SRB for 30 min, after which the excess dye was removed by washing repeatedly with 1% (vol/vol) acetic acid. The protein-bound dye was dissolved in 10 mM Tris base pH 8.8 solution for OD determination at 565-nm using a Varioskan™ LUX microplate reader (ThermoFisher). Note that Oligomycin reduces the viability of both MDA-MB-231 and MCF-10A cells, in a concentration-dependent manner at 120h (IC-50 ~ 5µM). Data represent the mean ± SD over, n=6. One-way ANOVA, Dunnett's multiple comparisons test, ****p* < 0.0005, *****p* < 0.0001.

**(D) Western blot analysis.** Effects of Bedaquiline 10µM and Oligomycin 5µM on ATP5F1C protein expression in MDA-MB-231 cells at 120 hours of treatment. ATP5F1C expression was not affected after 120h of Oligomycin (5µM) treatment compared with Bedaquiline (10µM) treatment (see also **Figure 7A**, for comparison). Vinculin was used as a control for equal protein loading.

**(E)** **ATP Levels.** Total ATP levels in MDA-MB-231 cells treated with Oligomycin (1, 2.5 and 5µM) were determined using Cell-Titer-Glo. After cell counting, 5 × 10^3^ cells were seeded in a white 96 well plate and then used to evaluate their relative ATP content by luminescence, using the Varioskan™ LUX plate reader. Note that, no differences were observed in ATP levels after Oligomycin treatment in MDA-MB-231 cells at 48, 72 and 120h cultured in DMEM high glucose media ^7, 8^. Measurements were normalized by Hoechst 33342 content (Excitation/Emission 350/461 nm). Data represent the mean fold change ± SD over control (vehicle alone; DMSO) cells, n=3. Two-way ANOVA, Sidak's multiple comparisons test.

**(F) Mitochondrial ROS Production.** MitoTracker® Orange CM-H_2_TMRos is a reduced, non-fluorescent version of MitoTracker Orange (M-7510) that fluoresces upon oxidation. Its accumulation is dependent upon membrane potential (Excitation/Emission 554/576 nm). CM-H_2_TMRos levels in MDA-MB-231 cells treated with Bedaquiline (5 and 10µM) and Oligomycin (2.5 and 5µM) were determined using the Attune NxT Flow Cytometer. Note that Bedaquiline 10µM increased the CM-H_2_TMRos signal in MDA-MB-231 cells already after 24 hours of treatment. In contrast, Oligomycin 5µM slightly increased the CM-H_2_TMRos signal only after 120 hours of treatment. The analysis was conducted on 25,000 cells. Data represent the mean fold change ± SD over control (vehicle alone; DMSO) cells, n=3. Two-way ANOVA, Sidak's multiple comparisons test. **p* < 0.05, ** *p* < 0.005, ****p* < 0.0005.

**Supplemental Figure S12. Bedaquiline does not target ATP5F1C protein expression or ATP production in MCF-10A cells, a non-tumourigenic breast cell line.**

**(A) Western blot analysis.** Effects of Bedaquiline (10 µM) on ATP5F1C protein expression in MCF-10A cells, after 72 and 120 hours of treatment. Note that no effects were detectable after 72 and 120 hours (see also Figure 7D); similarly, no effects were observed at earlier time points (24 and 48 hours; data not shown). β-tubulin and Vinculin were used as controls for equal protein loading. Antibodies and their dilutions used for Western blot analysis were as follows: mouse anti-ATP5F1C 1:500, mouse anti-β-tubulin 1:1,000, mouse anti-Vinculin 1:1,000.

**(B-C) Validation of ATP content.**

**(B)** Total ATP levels in MCF-10A cells were determined using Cell-Titer-Glo. After cell counting, equal numbers of single cells were then used to evaluate their relative ATP content by luminescence, using the Varioskan™ LUX plate reader. Note that, no differences were observed in ATP levels after Bedaquiline treatment in MCF-10A cells at 48, 72 and 120h. Measurements were normalized by Hoechst 33342 content (Excitation/Emission 350/461 nm). Data represent the mean fold change ± SD over control (vehicle alone; DMSO) cells, n=3. Two-way ANOVA, Sidak's multiple comparisons test.

**(C)** Mitochondrial ATP levels in MCF-10A cells were determined using Biotracker ATP-Red 1. After 30 minutes of incubation, the cells were analyzed using the Attune NxT Flow Cytometer. Note that Bedaquiline does not reduces the mitochondrial ATP levels in MCF-10A cells, at a concentration of 10 µM, in a time-dependent manner. The analysis was conduct on 25.000 cells. Data represent the mean fold change ± SD over control (vehicle alone; DMSO) cells, n=3. Two-way ANOVA, Sidak's multiple comparisons test, **p* < 0.05, ** *p* < 0.005, ****p* < 0.0005, *****p* < 0.0001.

**Supplemental Figure S13. Bedaquiline treatment of MDA-MB-231 cells specifically reduces the population of S-phase cells and increases cell death, in a dose- and time-dependent manner.**

**(A-C) Cell cycle analysis.** MDA-MB-231 cells were treated with Bedaquiline (1 and 10 μM) or vehicle alone, for 48, 72 and 120 hours, and then subjected to cell cycle analysis by FACS. Note that no effects were detectable at 48 hours. However, at 72 and 120 hours, we observed a decrease in the S-phase population and a concomitant increase in the sub-G0-G1 population. Two-way ANOVA, Sidak's multiple comparisons test, ns=not significant, *p< 0.01, **p< 0.001, ***p < 0.0005.

**(D-F) Live/dead analysis.** MDA-MB-231 cells were treated with Bedaquiline (1 and 10 μM) or vehicle alone, for 48, 72 and 120 hours, and then subjected to live/dead analysis by FACS. Note that no effects were detectable at 48 hours. However, at 72 and 120 hours, we observed a decrease in the live cell population and an increase in the dead cell population. However, no increase in the apoptotic cell population (early or late) was noted, suggesting that cell death was due to necrosis. Two-way ANOVA, Sidak's multiple comparisons test, ns=not significant, *p<0.01, **p<0.001, ***p< 0.0005.

**(G)** **Western blot analysis.** Effects of Bedaquiline (0, 0.1, 1 and 10 μM) on PARP and p21 protein expression in MDA-MB-231 cells, after 120 hours of treatment. Note that PARP and p21 decreased in a concentration-dependent manner. Beta-actin was used as a control for equal protein loading. Note: The Beta-actin shown here is the same as in **Figure 7, panel A,** at 120 hours, because this blot was stripped and re-probed for PARP and p21 expression.

**Supplemental Figure S14. Bedaquiline treatment of MDA-MB-231 cells induces autophagy, in a time-dependent manner.**

**(A) Flow Cytometry analysis of LC3 antibody**. The autophagy Induction ratio was calculated using the Autophagy LC3-antibody based Kit for Guava® Muse® Cell Analyzer by Luminex. MDA-MB-231 cells were treated with Bedaquiline (10 µM) or vehicle alone, for 48, 72 and 120 hours, and then subjected to analysis by flow cytometry. Note that no effects were detectable at 24 hours. However, at 48, 72 and 120 hours, we observed an increase of the Autophagy Induction Ratio in Bedaquiline-treated cells, based on the LC3 intensity of the signal. The analysis was conducted on 10,000 cells. Data represent the mean fold change ± SD over control (vehicle alone; DMSO) cells, n=3. Two-way ANOVA, Sidak's multiple comparisons test, **p< 0.005, ****p < 0.0001.

**(B)** **Western blot analysis.** Effects of Bedaquiline (10 µM) on SQSTM1/p62, LC3B type I/II and Phospho-Ubiquitin (Ser 65) protein expression in MDA-MB-231 cells, after 72 and 120 hours of treatment. Note that SQSTM1/p62 and LC3B type II expression increased in Bedaquiline treated cells. Moreover, Phospho-Ubiquitin (Ser65) expression was highly increased at 120h of treatment. Vinculin was used as a control for equal protein loading. Antibodies and their dilutions used for Western blot analysis were as follows: rabbit anti-SQSTM1/p62 1:1,000, rabbit anti-LC3B 1:1,000, rabbit anti-Phospho-Ubiquitin 1:1,000, mouse anti-Vinculin 1:1,000.

**Supplemental Reagent Information**

**Antibody List**

**Name Catalogue Number Source___________**

Anti-ATP5F1C IgG ab119686 (ATP5C1) ABCAM

Anti-ATP5F1C IgG ab241076 (ATP5C1) ABCAM

Anti-HSP60 (H-1) IgG sc-13115 Santa Cruz Biotech

Anti-Total OXPHOS IgG ab110411 ABCAM (Cocktail Abs)

Anti-HIF-1α IgG ab1[H1alpha67] ABCAM

Anti-EpCAM (C-10) IgG sc-25308 Santa Cruz Biotech

Anti-VCAM1 (E-10) IgG sc-13160 Santa Cruz Biotech

Anti- β-Tubulin (D-10) IgG sc-5274 Santa Cruz Biotech

Anti-p21 (CP74) IgG MA5-1453 Invitrogen

Anti-β-Actin IgG A2228 Sigma-Aldrich

Anti-SQSTM1/p62 (D5E2) IgG #8025 Cell Signaling

Anti-Vinculin (H-10) IgG sc-25336 Santa Cruz Biotech

Anti-PARP (46D11) IgG #9532 Cell Signaling

Anti-LC3B (D11) IgG #3868 Cell Signaling

Anti-Phospho-Ubiquitin (Ser65) IgG sc-25336 Santa Cruz Biotech

Anti-Mouse IgG 7076S Cell Signaling

Anti-Rabbit IgG 7074P Cell Signaling

**Construct List**

**Name Catalogue Number Source___________**

shRNA-ATP5F1C CS-HSH103401-LVRInU6TGP-01 GeneCopoeia

An shRNA clone set of 3 constructs (designated a, b, c) against all 3 variants for human ATP5F1C in the lentiviral psi-LVRInU6TGP vector, with an inducible U6 promoter, CMV promoter-TetR-SV40 promoter-eGFP-IRES-puromycin.

shRNA-Control CS-HCTR001-LVRInU6TGP GeneCopoeia

A scrambled control for the above psi-LVRInU6TGP vector.

**Supplemental References**

1. Yotsumoto F, Tokunaga E, Oki E, Maehara Y, Yamada H, Nakajima K*, et al.* Molecular hierarchy of heparin-binding EGF-like growth factor-regulated angiogenesis in triple-negative breast cancer. *Mol Cancer Res* 2013, **11**(5)**:** 506-517.

2. Reyngold M, Turcan S, Giri D, Kannan K, Walsh LA, Viale A*, et al.* Remodeling of the methylation landscape in breast cancer metastasis. *PLoS One* 2014, **9**(8)**:** e103896.

3. Wang Y, Klijn JG, Zhang Y, Sieuwerts AM, Look MP, Yang F*, et al.* Gene-expression profiles to predict distant metastasis of lymph-node-negative primary breast cancer. *Lancet* 2005, **365**(9460)**:** 671-679.

4. Parikh H, Nilsson E, Ling C, Poulsen P, Almgren P, Nittby H*, et al.* Molecular correlates for maximal oxygen uptake and type 1 fibers. *Am J Physiol Endocrinol Metab* 2008, **294**(6)**:** E1152-1159.

5. Rivera-Torres J, Acin-Perez R, Cabezas-Sanchez P, Osorio FG, Gonzalez-Gomez C, Megias D*, et al.* Identification of mitochondrial dysfunction in Hutchinson-Gilford progeria syndrome through use of stable isotope labeling with amino acids in cell culture. *J Proteomics* 2013, **91:** 466-477.

6. Fina E, Callari M, Reduzzi C, D'Aiuto F, Mariani G, Generali D*, et al.* Gene expression profiling of circulating tumor cells in breast cancer. *Clin Chem* 2015, **61**(1)**:** 278-289.

7. Palorini R, Simonetto T, Cirulli C, Chiaradonna F. Mitochondrial complex I inhibitors and forced oxidative phosphorylation synergize in inducing cancer cell death. *Int J Cell Biol* 2013, **2013:** 243876.

8. Lunetti P, Di Giacomo M, Vergara D, De Domenico S, Maffia M, Zara V*, et al.* Metabolic reprogramming in breast cancer results in distinct mitochondrial bioenergetics between luminal and basal subtypes. *FEBS J* 2019, **286**(4)**:** 688-709.
